# Essential role of hydride ion in ruthenium-based ammonia synthesis catalysts†

**DOI:** 10.1039/c6sc00767h

**Published:** 2016-04-21

**Authors:** Masaaki Kitano, Yasunori Inoue, Hiroki Ishikawa, Kyosuke Yamagata, Takuya Nakao, Tomofumi Tada, Satoru Matsuishi, Toshiharu Yokoyama, Michikazu Hara, Hideo Hosono

**Affiliations:** a Materials Research Center for Element Strategy, Tokyo Institute of Technology 4259 Nagatsuta, Midori-ku Yokohama 226-8503 Japan hosono@msl.titech.ac.jp; b Laboratory for Materials and Structures, Tokyo Institute of Technology 4259 Nagatsuta, Midori-ku Yokohama 226-8503 Japan mhara@msl.titech.ac.jp; c ACCEL, Japan Science and Technology Agency 4-1-8 Honcho, Kawaguchi Saitama 332-0012 Japan; d Frontier Research Center, Tokyo Institute of Technology 4259 Nagatsuta, Midori-ku Yokohama 226-8503 Japan

## Abstract

The efficient reduction of atmospheric nitrogen to ammonia under low pressure and temperature conditions has been a challenge in meeting the rapidly increasing demand for fertilizers and hydrogen storage. Here, we report that Ca_2_N:e^−^, a two-dimensional electride, combined with ruthenium nanoparticles (Ru/Ca_2_N:e^−^) exhibits efficient and stable catalytic activity down to 200 °C. This catalytic performance is due to [Ca_2_N]^+^·e_1−*x*_^−^H_*x*_^−^ formed by a reversible reaction of an anionic electron with hydrogen (Ca_2_N:e^−^ + *x*H ↔ [Ca_2_N]^+^·e_1−*x*_^−^H_*x*_^−^) during ammonia synthesis. The simplest hydride, CaH_2_, with Ru also exhibits catalytic performance comparable to Ru/Ca_2_N:e^−^. The resultant electrons in these hydrides have a low work function of 2.3 eV, which facilitates the cleavage of N_2_ molecules. The smooth reversible exchangeability between anionic electrons and H^−^ ions in hydrides at low temperatures suppresses hydrogen poisoning of the Ru surfaces. The present work demonstrates the high potential of metal hydrides as efficient promoters for low-temperature ammonia synthesis.

## Abstract 2

Ruthenium-loaded metal hydrides with hydrogen vacancies function as efficient catalysts for ammonia synthesis under low temperature and low pressure conditions.

## Graphical abstract

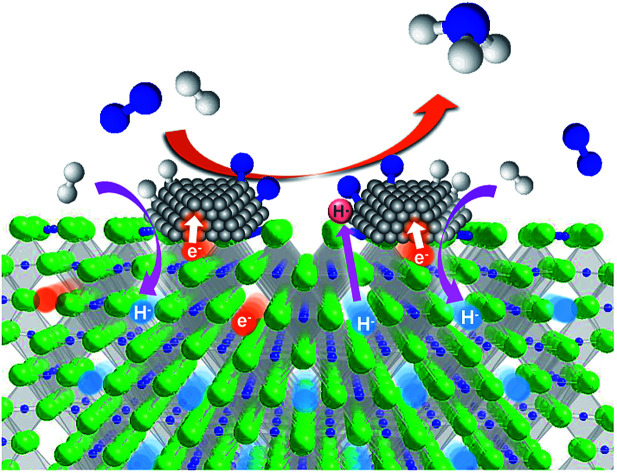

## Introduction

Catalytic ammonia synthesis is vital for the production of synthetic fertilizers and serves as an active nitrogen source for important chemicals. Dinitrogen (N_2_) is a typically inert molecule because of the strong N≡N bond (945 kJ mol^−1^).^1^ Therefore, high temperature (400–600 °C) and high pressure (20–40 MPa) are required for industrial ammonia synthesis (Haber–Bosch process),^2^ which results in high energy consumption. Recently, ammonia has also attracted much attention as a hydrogen storage material due to its high capacity for hydrogen storage (17.6 wt%) and facile liquefaction under mild conditions.^3^ Ammonia synthesis at low temperature is thermodynamically favorable but still presents a major challenge. Extensive studies on N_2_ activation with organometallic complexes have been conducted over the last decade.^4–7^ Although NH_3_ has been successfully produced under ambient conditions, the rate of formation is still far from appropriate for practical application, and strong reducing agents and extra proton sources are required to afford NH_3_.

In heterogeneous catalysts, it is widely recognized that ruthenium (Ru) catalysts work under milder conditions than iron-based catalysts for the Haber–Bosch process.^8,9^ The activity of Ru catalysts is substantially enhanced by electron injection from alkali or alkali earth metal oxide promoters.^8,10^ Although these electronic promoters lower the energy barrier for N_2_ dissociation, the enthalpy of hydrogen adsorption on the Ru catalyst is also increased, leading to high surface coverage by H atoms (hydrogen poisoning).^11^ Accordingly, the electronic promotion effect for N_2_ dissociation is retarded by the competitive adsorption of H_2_. It is therefore highly desirable to develop a new Ru catalyst that can promote N_2_ dissociation and prevent hydrogen poisoning. It was demonstrated that the 12CaO·7Al_2_O_3_ electride (C12A7:e^−^)^12^-supported Ru catalyst exhibits much higher activity for ammonia synthesis than alkali-promoted Ru catalysts.^13,14^ The intrinsically low work function (*ca.* 2.4 eV) of C12A7:e^−^^15^ in this catalyst promotes N_2_ dissociation on Ru, which leads to a reduction in the activation energy to half (*ca.* 55 kJ mol^−1^) of that for conventional Ru catalysts. A recent kinetic analysis revealed that the bottleneck for ammonia synthesis is shifted from N_2_ dissociation to the formation of N–H_*n*_ species.^16^ In addition, this catalyst has reversible exchangeability of electrons and hydride ions, and is almost immune to hydrogen poisoning of the Ru surface, which is a serious drawback for conventional Ru catalysts. These results imply that both electrons and hydride ions play a crucial role in effective ammonia synthesis. However, the outstanding activity of C12A7:e^−^ is diminished at low temperatures (<320 °C), which is strongly correlated with the weak H_2_ desorption properties at low temperatures. The electron–hydride ion exchange reaction in C12A7:e^−^ is accomplished by H desorption through a cage wall composed of a rigid monolayer of Ca–Al–O; therefore, the exchange reaction in C12A7:e^−^ requires a relatively high temperature that is sufficient to excite thermal vibration and allow H to escape from the cage. Therefore, our design concept for a highly active low-temperature ammonia synthesis catalyst is embodied by inorganic electride materials with hydride ions exposed to the surface, *i.e.*, metal hydrides.

Dicalcium nitride, [Ca_2_N]^+^·e^−^ (denoted as Ca_2_N:e^−^), was confirmed as a two-dimensional (2D) electride with a low work function (2.6 eV), in which anionic electrons confined between the [Ca_2_N]^+^ layers as counter anions^17–19^ can be partly exposed to the surfaces. In addition, this material can be converted into Ca_2_NH ([Ca_2_N]^+^·H^−^) by the reaction between an anionic electron and a hydrogen, which is analogous to that for C12A7:e^−^. Therefore, Ca_2_NH was selected as the first test bed material to verify our hypothetical design concept of metal hydrides for low-temperature ammonia synthesis.

Here we report that metal hydride materials such as Ca_2_NH and CaH_2_, which are not intrinsically low work function materials, strongly promote the cleavage of N_2_ to form NH_3_ on Ru nanoparticles under low pressure and temperature conditions. The low work function (2.3 eV) is immediately realized by the formation of hydrogen vacancies in these Ru-loaded hydride materials during ammonia synthesis, which in turn facilitates N_2_ dissociation and prevents hydrogen poisoning of the Ru surface.

## Results and discussion

### Catalytic performance of Ru-loaded Ca_2_N:e^−^

**Fig. 1:**
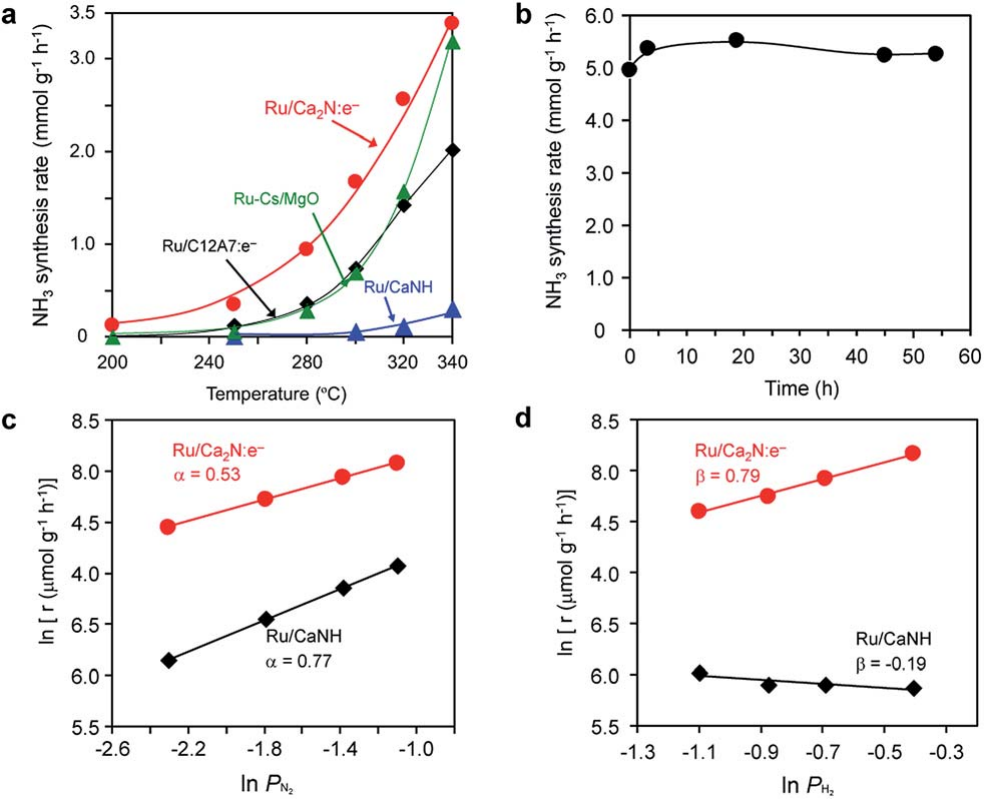
(a) Catalytic activity for ammonia synthesis over various Ru catalysts (2 wt%) as a function of reaction temperature (reaction conditions: catalyst, 0.1 g; WHSV, 36 000 mL g_cat_^−1^ h^−1^; reaction pressure, 0.1 MPa). (b) Reaction time profile for ammonia synthesis over Ru (5 wt%)/Ca_2_N at 340 °C (reaction conditions: catalyst, 0.1 g; WHSV, 36 000 mL g_cat_^−1^ h^−1^; reaction pressure, 1.0 MPa). (c, d) Dependence of ammonia synthesis on the partial pressure of (c) N_2_ and (d) H_2_ using various Ru catalysts (2 wt%) at 340 °C under atmospheric pressure.

Fig. 1 shows the temperature dependence for ammonia synthesis over various Ru catalysts and Table 1 summarizes the catalytic properties of various Ru catalysts. The Ru dispersion of Ru/Ca_2_N:e^−^ (3.1%) is much smaller than that of Ru(2%)–Cs/MgO (50.4%) because of its low surface area (1.5 m^2^ g^−1^), which is similar to the results for Ru/C12A7:e^−^. However, Ru/Ca_2_N:e^−^ exhibits high catalytic activity (at 340 °C) comparable to Ru(2%)–Cs/MgO, which is one of the most active catalysts for ammonia synthesis reported to date.^20,21^ Accordingly, the turnover frequency (TOF) with Ru/Ca_2_N:e^−^ is higher than that with Ru–Cs/MgO by an order of magnitude. It is noteworthy that Ru/Ca_2_N:e^−^ exhibits higher catalytic activity than Ru/C12A7:e^−^ or Ru(2%)–Cs/MgO, especially below 300 °C, and ammonia formation can be distinctly observed even at 200 °C. The apparent activation energy of Ru/Ca_2_N:e^−^ for ammonia synthesis is 60 kJ mol^−1^, which is one-half that of Ru–Cs/MgO (120 kJ mol^−1^). We have previously reported^16^ that a change in the activation energy is observed for Ru/C12A7:e^−^, *i.e.*, the activation energy at 400–320 °C is 50 kJ mol^−1^ but exceeds 90 kJ mol^−1^ at lower reaction temperatures (320–250 °C) and no reaction is observed at 200 °C. These results indicate that Ru/Ca_2_N:e^−^ is superior to Ru/C12A7:e^−^ for low-temperature ammonia synthesis. Furthermore, we note that Ru/Ca_2_N:e^−^ functions as a stable catalyst for ammonia synthesis over long periods without degradation in activity. Fig. 1b shows that the initial ammonia synthesis rate was maintained even after 54 h under high pressure conditions (1.0 MPa), and the total amount of produced ammonia reached 27 mmol, which is more than 25 times the total nitrogen content in Ca_2_N:e^−^ (1.06 mmol). This result indicates that the ammonia produced is not derived from the decomposition of the Ca_2_N:e^−^ support.

**Table 1:** Catalytic properties of various Ru catalysts

Catalyst	Ru loading (wt%)	*S*_BET_ (m^2^ g^−1^)	*D*_m_ a (%)	*d* a (nm)	*N*_S_ a (mmol g^−1^)	*r*_NH_3__ b (mmol g^−1^ h^−1^)		TOF^c^ (×10^3^) (s^−1^)		*E*_a_ d (kJ mol^−1^)
						340 °C	300 °C	340 °C	300 °C	
Ru/Ca_2_N:e^−^	1.8	1.5	3.1	42.8	5.1	3386	1674	185.1	91.5	60
Ru/CaNH	1.8	1.0	4.5	30.2	7.2	308	53	11.9	2.0	110
Ru/C12A7:e^−^	1.8	1.0	4.7	28.7	8.3	2021	745	67.5	24.9	51 (400–320 °C)
										91 (320–200 °C)
Ru/CaH_2_	2.0	3.8	12.3	10.9	22.3	4002	2549	153.9	98.0	51
Ru–Cs/MgO	2.0	12.0	50.4	2.5	45.7	3200	697	19.5	4.2	120

a Dispersion (*D*_m_), particle size (*d*), and the number of surface Ru atoms (*N*_S_) were calculated on the basis of CO chemisorption values, assuming spherical metal particles and a stoichiometry of Ru/CO = 1.^37^

b NH_3_ synthesis rate (*r*_NH_3__); conditions: catalyst (0.1 g), synthesis gas (H_2_/N_2_ = 3, 60 mL min^−1^), weight hourly space velocity (WHSV) = 36 000 mL g_cat_^−1^ h^−1^, pressure (0.1 MPa).

c TOF was calculated from the rate of ammonia synthesis divided by *N*_S_.

d *E*_a_ is the apparent activation energy calculated from Arrhenius plots for the reaction rate in the temperature range of 340–250 °C.

Fig. 1c and d show the dependence of the ammonia synthesis rate on the partial pressure of N_2_ and H_2_, respectively. The reaction orders with respect to N_2_, H_2_, and NH_3_ over various Ru catalysts are also summarized in Table S1.† The reaction order for N_2_ with conventional heterogeneous catalysts is 0.8–1.0,^11,20,21^ where N_2_ dissociation is the rate-determining step for ammonia synthesis. In contrast, the reaction order for N_2_ with Ru/Ca_2_N:e^−^ is almost one-half, which is attributed to a more dense population of N adatoms on Ru/Ca_2_N:e^−^ than on the other catalysts. Two results were noted; one is that the Ru/Ca_2_N:e^−^ catalyst also facilitates the N_2_ isotopic exchange reaction (^15^N_2_ + ^14^N_2_ = 2^15^N^14^N) with a lower activation energy (59 kJ mol^−1^) than conventional Ru catalysts (>130 kJ mol^−1^)^16^ (Fig. S1†), indicating that the energy barrier for N_2_ dissociation is significantly lowered by Ru/Ca_2_N:e^−^. The other result is that Ru/Ca_2_N:e^−^ has a positive reaction order with respect to H_2_, in contrast to the case for Ru–Cs/MgO (*β* < 0), which indicates that ammonia synthesis over Ru/Ca_2_N:e^−^ is not inhibited by hydrogen adsorption, *i.e.*, hydrogen poisoning.^11,20^ Thus, Ru/Ca_2_N:e^−^ maintains the key kinetics observed with Ru/C12A7:e^−^, in that N_2_ cleavage is not the rate-determining step for ammonia synthesis^16^ and the reactions are free from hydrogen poisoning on the Ru surface. Generally, the promotion effect of alkali compounds such as Cs-oxide in a Ru catalyst is a trade-off between lowering the activation barrier for N_2_ dissociation and increasing the competitive adsorption of H_2_.^11^ However, this serious drawback can be overcome by using Ca_2_N:e^−^ as a support material. Furthermore, the catalytic activity of Ru/Ca_2_N:e^−^ increased with an increase in the reaction pressure at 320 °C (Fig. S2†). On the other hand, the increment in the catalytic activity of Ru/C12A7:e^−^ is moderate at this temperature, which is due to the poisoning effect of H atoms on Ru/C12A7:e^−^ at low reaction temperature (≤320 °C).^16^ These results clearly indicate that Ru/Ca_2_N:e^−^ exhibits improved performance for ammonia synthesis compared with Ru/C12A7:e^−^, even at lower temperatures and elevated pressures.

### Structural properties of Ca-nitride catalysts

**Fig. 2:**
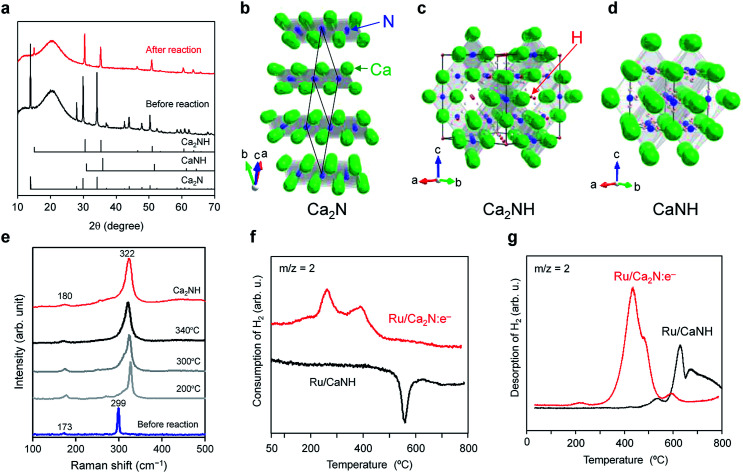
(a) XRD patterns for Ru/Ca_2_N:e^−^ before and after ammonia synthesis reaction at 340 °C for 20 h. Standard JCPDS diffraction patterns for Ca_2_N (space group *R*3̄*m*, PDF: 70-4196), CaNH (space group *Fm*3̄*m*, PDF: 75-0430), and Ca_2_NH (space group *Fd*3̄*m*, PDF: 76-608) are provided for reference. (b–d) Crystal structures of Ca_2_N (b), Ca_2_NH (c), and CaNH (d) were visualized using the VESTA program.^38^ (b) Ca_2_N:e^−^ has a hexagonal layered structure with anionic electron layers between the cationic framework layers ([Ca_2_N]^+^) composed of edge-sharing NCa_6_ octahedra. (c) Ca_2_NH is composed of Ca^2+^, N^3−^, and H^−^ ions, where Ca atoms form a slightly distorted cubic close packed structure, and N and H are ordered in each anion layer. (d) CaNH, an inorganic imide compound with a cubic structure, consists of Ca^2+^ and NH^2−^ ions. (e) *In situ* Raman spectra for Ru/Ca_2_N:e^−^ measured under ammonia synthesis conditions (N_2_ : H_2_ = 1 : 3, 0.1 MPa, 60 mL min^−1^). The Raman spectrum for Ca_2_NH is also shown as a reference. (f) H_2_ TPA profiles for Ru/Ca_2_N:e^−^ and Ru/CaNH catalysts. The TPA measurements were performed (1 °C min^−1^) with a dilute mixture of H_2_ (5%) in Ar. (g) H_2_ TPD profiles for Ru/Ca_2_N:e^−^ and Ru/CaNH after ammonia synthesis reaction at 340 °C for 10 h. The TPD measurements were performed (1 °C min^−1^) under Ar flow.

X-ray diffraction (XRD) and Raman spectroscopy measurements confirmed that Ca_2_NH, an inorganic hydride, is formed in the Ru/Ca_2_N:e^−^ catalyst during the ammonia synthesis reaction. Fig. 2a shows that Ru/Ca_2_N:e^−^ after ammonia synthesis has no diffraction peaks attributable to the Ca_2_N phase (Fig. 2b),^19,22,23^ whereas new peaks due to Ca_2_NH with cubic structure (Fig. 2c) appear.^23–25^ There is no CaNH phase (cubic structure, Fig. 2d), which consists of Ca^2+^ and NH^2−^ ions (the formal charge of hydrogen is +1), in Ru/Ca_2_N:e^−^ after the reaction.^26–28^ The formation of Ca_2_NH in the catalyst was also elucidated from *in situ* Raman spectroscopy measurements (Fig. 2e). Two intense bands at 173 and 299 cm^−1^ for Ca_2_N:e^−^ are red-shifted to 180 and 322 cm^−1^, respectively, under the reaction conditions. The Raman spectrum for CaNH has broad bands in the range from 100 to 1000 cm^−1^ and is completely different from that of Ca_2_NH (Fig. S3†). In addition, CaNH has an intense band centered at 3122 cm^−1^, which is attributed to the N–H stretching mode in imide ions.^29,30^ Although Ru/Ca_2_N:e^−^ also showed weak bands in the range of 3100–3300 cm^−1^ after the reaction, the intensity is much smaller than that for CaNH, which indicates that the catalyst consists mainly of Ca_2_NH. In addition, Ru/CaNH has much lower catalytic activity and a higher activation energy (110 kJ mol^−1^) than Ru/Ca_2_N:e^−^ (Fig. 1, S2† and Table 1). The kinetic analysis (Fig. 1 and Table S1†) revealed that N_2_ cleavage is the rate-determining step for Ru/CaNH and the catalyst is subject to hydrogen poisoning like the conventional Ru catalysts.

To understand the reactivity of these materials with hydrogen, temperature-programmed absorption (TPA) and desorption (TPD) of H_2_ on the catalysts were examined. Ru/Ca_2_N:e^−^ can absorb hydrogen above 150 °C, which means that the hydrogen storage reaction (H^0^ + e^−^ → H^−^) takes place to form Ca_2_NH (Fig. 2f). In contrast, no H_2_ absorption peak was observed for Ru/CaNH and a negative peak appeared at 500–600 °C, which corresponds to hydrogen desorption from the sample, *i.e.*, decomposition. Fig. 2g shows that H_2_ is released (H^−^ → H^0^ + e^−^) from Ru/Ca_2_N:e^−^ after reaction above 200 °C (the actual material is Ru/Ca_2_NH) and the H^−^ ion content was estimated to be 10.6 mmol g^−1^, which is in good agreement with the theoretical amount (10.5 mmol g^−1^) of H^−^ ions in Ca_2_NH. Furthermore, the onset temperature for H_2_ desorption from Ru/Ca_2_N:e^−^ after the reaction is much lower than that for Ru/CaNH. The TPA and TPD results show that hydrogen incorporation and desorption reactions proceed above 200 °C over Ru/Ca_2_N:e^−^. Therefore, a nonstoichiometric phase represented by [Ca_2_N]^+^·e_1−*x*_^−^H_*x*_^−^, rather than stoichiometric Ca_2_NH, is formed by the reaction between anionic electrons and H^−^ ions (Ca_2_N:e^−^ + H ↔ [Ca_2_N]^+^·H^−^) during ammonia synthesis. This exchangeability is a key reaction confirmed in Ru/C12A7:e^−^, but the significant difference between Ru/Ca_2_N:e^−^ and Ru/C12A7:e^−^ is the onset temperature for H_2_ desorption, *i.e.*, 200 °C for Ru/Ca_2_N:e^−^ and 350 °C for Ru/C12A7:e^−^.

### DFT calculations

**Fig. 3:**
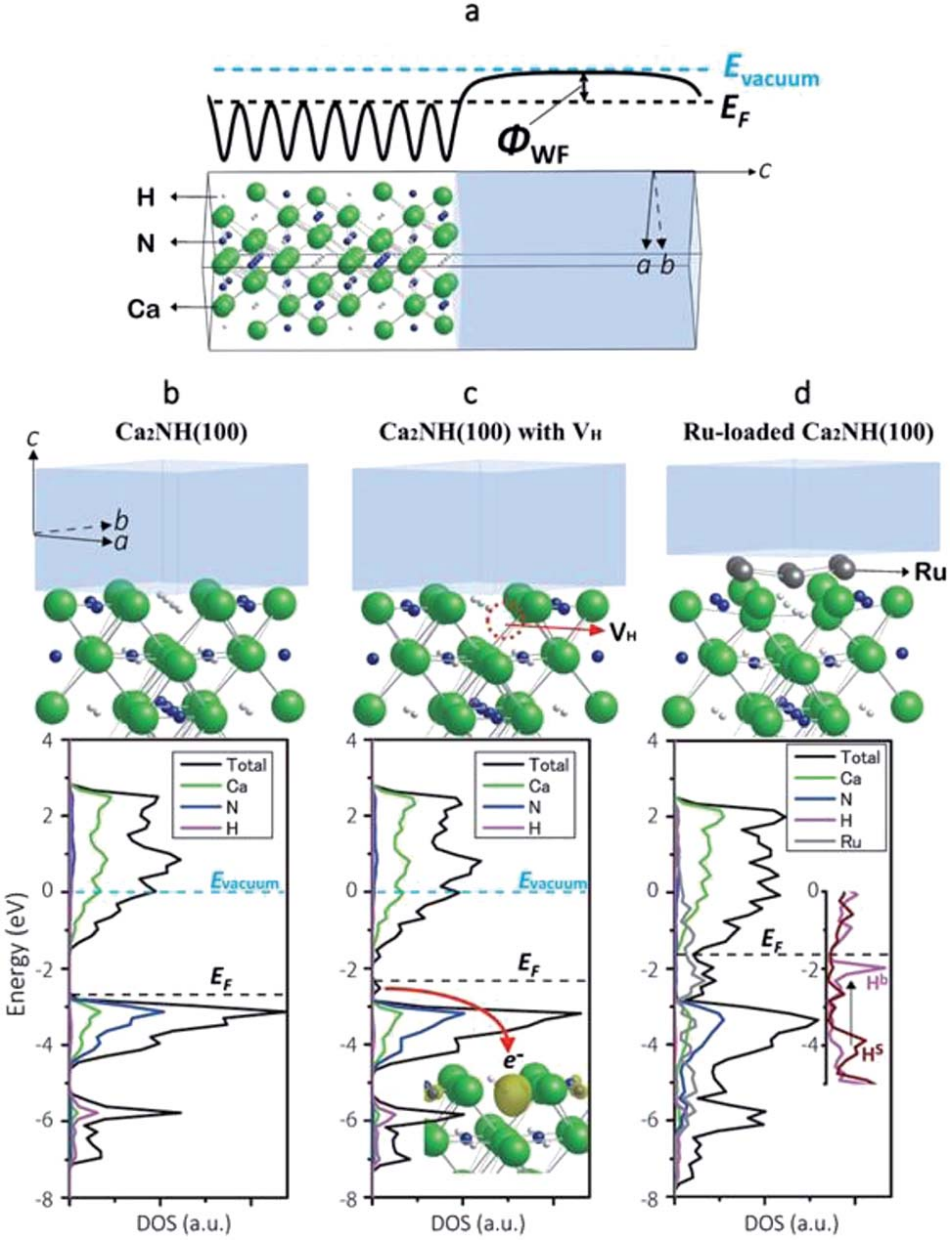
(a) Computational model used in the calculation of the work function of Ca_2_NH(100). The vacuum region (transparent gray) is included in the model to determine the vacuum level from the electrostatic potential profile (solid thick line) in the region. (b) Surface structure and spin-averaged DOS of Ca_2_NH(100), where an energy of 0.0 eV corresponds to the vacuum level. (c) Surface structure and spin-averaged DOS of Ca_2_NH(100) with a hydrogen vacancy (V_H_), indicated with a red dotted circle. The energy of 0.0 eV corresponds to the vacuum level. Inset: the local density (yellow) of anionic electron states just below *E*_F_ (a red arrow) depicted with an isosurface value of 0.015 e^−^ bohr^−3^. The anionic electron states are purely spin polarized states. (d) Surface structure and spin-averaged DOS of Ru-loaded Ca_2_NH(100). The vacuum level was not determined in this model; therefore, the DOS were represented to match the VBM of nitrogen with those of Ca_2_NH(100) with/without V_H_. Inset: DOS of H bonded with Ru(H^b^) and H on the free surface (H^s^). Green, blue, white, and gray atoms in the atomistic models correspond to Ca, N, H, and Ru, respectively. These crystal structures and charge distributions were visualized using the VESTA program.^38^

To shed more light on the electronic state of Ca_2_NH, density functional theory (DFT) calculations were conducted (detailed conditions are described in the ESI†). Fig. 3a shows a computational model of the Ca_2_NH(100) surface, and Fig. 3b–d show the calculated electronic states of Ca_2_NH(100), Ca_2_NH_1−*x*_(100), and Ru-loaded Ca_2_NH(100), respectively. The calculations were executed under the condition of an electrically neutral unit cell. The calculated work functions (WFs)^31^ for Ca_2_N (3.3, 2.5 eV) and Ru (4.8 eV) are close to the measured values^19,32,33^ (Table 2), which indicates the reliability of the DFT calculations. The calculated WF for Ca_2_NH is significantly smaller than that for CaNH, which indicates the higher electron-donating ability of Ca_2_NH than CaNH. Notably, Ca_2_NH(100) with a hydrogen vacancy (V_H_) (*i.e.*, Ca_2_NH_1−*x*_(100)) shows a much lower WF (2.3 eV) than both Ca_2_NH (2.8 eV) and Ca_2_N:e^−^ (2.5 eV). When an anionic electron is confined at the V_H_ position in Ca_2_NH (see the inset of Fig. 3c), the anionic electron state is located above the valence band maximum (VBM) of Ca_2_NH, as shown in Fig. 3c. The confinement of the anionic electron in Ca_2_NH_1−*x*_(100) is stronger than that of the 2D layered space in Ca_2_N, and thus Ca_2_NH(100) with V_H_ has a small WF, which reflects the strong electron-donation ability of Ca_2_NH_1−*x*_.

**Table 2:** Calculated work functions and hydrogen vacancy (V_H_) formation energies^a^

Compound	Space group	Surface index	WF^calc^ (eV)	WF^exp^ (eV)	Δ*E*(V_H_) (eV per atom)
Ca_2_N:e^−^	*R*3̄*m*	(111)	3.3	3.5^b^	—
Ca_2_N:e^−^	*R*3̄*m*	(112)	2.5	2.6^b^	—
Ca_2_NH	*Fd*3̄*m*	(100)	2.8^c^	—	—
Ca_2_NH_1–1/16_	*Fd*3̄*m*	(100)	2.3	—	1.00
Ca_2_NH_1–2/16_	*Fd*3̄*m*	(100)	2.3	—	0.88–1.02
Ru/Ca_2_NH_1–1/16_	—	(100)	—	—	0.43–0.67
Ru/N_2_/Ca_2_NH_1–1/16_	—	(100)	—	—	0.49–0.91
CaH_2_	*Pnma*	(010)	4.0	—	—
CaH_2–1/24_	*Pnma*	(010)	2.3	—	1.08–1.11
CaNH	*Fm*3̄*m*	(100)	≥3.6^c^^,^^d^	—	—
CaNH_1–1/32_	*Fm*3̄*m*	(100)	—	—	1.71

a Δ*E*(V_H_) for compound AH_1−*x*_ is the total energy difference, defined as [*E*(AH_1−*x*_) + *xE*(H_2_)/2] − *E*(AH), where *E*(AH), *E*(AH_1−*x*_), and *E*(H_2_) are, respectively, the total energies of the stoichiometric AH, H-deficient AH_1−*x*_, and hydrogen molecule.

b Ref. 13.

c Ca_2_NH and CaNH are not metallic compounds, and thus the values of WF^calc^ correspond to the positions of the valence band maximum with respect to the vacuum level.

d The H atoms in CaNH show partial occupation, so that determination of the hydrogen positions for DFT calculations is a difficult task; therefore, the following assumptions were adopted only for CaNH: that (1) the lattice parameters are fixed to the experimental values, (2) the positions of hydrogen were determined to maintain the local stoichiometry at the topmost layer of the CaNH surface, and (3) the ionic positions were all fixed to those in the bulk to avoid an artificial surface reconstruction caused by an ordered configuration of H atoms. If the surface structure of CaNH with the ordered configuration of hydrogen is relaxed, then the vacuum level required for the WF calculation cannot be determined. In a realistic situation of CaNH, ionic relaxations lead to a more stable electronic structure, and thereby the position of the valence band maximum becomes deeper. Therefore, the calculated value based on the above assumptions can be recognized as a lower limit.

Ca_2_NH_1−*x*_ can be readily formed by the combination of Ca_2_NH with Ru nanoparticles. Table 2 lists the calculated formation energies of V_H_ on the surfaces of the catalysts in the presence or absence of Ru. Relatively large V_H_ formation energies (0.88–1.02 eV) were obtained for Ru-free Ca_2_NH(100), which indicates the difficulty of hydrogen vacancy formation. The density of states (DOS) for an H^−^ ion in Ca_2_NH(100) is found at a relatively deep level, as shown in Fig. 3c and the inset of Fig. 3d (−6 eV below the Fermi level (*E*_F_) for bulk H^−^ and −4 eV below *E*_F_ for surface H^−^); therefore, the formation of anionic electrons by H desorption requires a relatively large amount of energy. However, the situation is significantly changed when a Ru cluster is loaded on the Ca_2_NH(100) surface; the V_H_ formation energy for Ru_6_-loaded Ca_2_NH(100) is decreased to 0.43–0.67 eV. Fig. 3d shows that the DOS for an H^−^ ion bonded with Ru (H^b^ in the inset of Fig. 3d) are energetically shifted up from a relatively deep level to a shallow level (−0.5 eV below *E*_F_). Thus, an H^−^ ion bonded with Ru is almost ready to form an anionic electron by the desorption of H^0^. The lift-up of the H^−^ states is caused by the fraction of the anti-bonding level with Ru, and thus originates from the presence of surface H^−^. In other words, the orbital hybridization between Ru and surface H^−^ leads to an electron donation from surface H^−^ to Ru, which makes the desorption as H^0^ easier. This situation is maintained even when an N_2_ molecule is adsorbed on Ru; the formation energy for V_H_ on Ru_6_N_2_–Ca_2_NH(100) is 0.49 eV. In contrast, a large V_H_ formation energy is observed for CaNH (1.71 eV), which corresponds well to the experimental evidence presented in Fig. 2g. These computational results support the idea that anionic electrons with a low WF are formed in nonstoichiometric hydrides, [Ca_2_N]^+^·e_1−*x*_^−^H_*x*_^−^, by Ru catalysts during the reaction, which facilitates N_2_ cleavage on the hydride *via* electron donation from the anionic electrons to the loaded Ru nanoparticles.

### Reaction mechanism over Ru/[Ca_2_N]^+^·e_1−*x*_^−^H_*x*_^−^

**Fig. 4:**
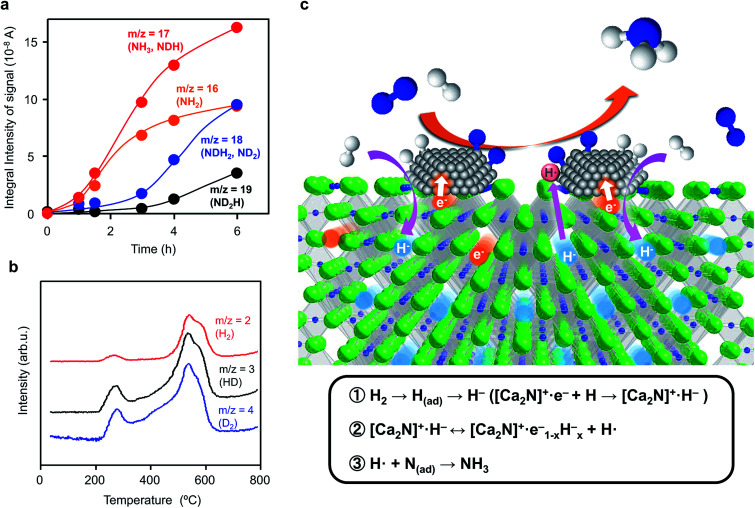
(a) Reaction time profiles for ammonia synthesis from N_2_ and D_2_ over Ru/Ca_2_N:e^−^ at 340 °C (reaction conditions: catalyst, 0.2 g; reaction gas, N_2_ : D_2_ = 1 : 3; reaction pressure, 60 kPa). Prior to reaction, Ru/Ca_2_N:e^−^ was heated under N_2_ + H_2_ flow (N_2_ : H_2_ = 1 : 3) at 340 °C for 10 h to form Ru/Ca_2_NH. (b) TPD profiles of Ru/Ca_2_N:e^−^ after the reaction (a). TPD measurements were performed (10 °C min^−1^) with Ar flow. (c) Schematic illustration of ammonia synthesis over Ru/Ca_2_N:e^−^. During ammonia synthesis over Ru/Ca_2_N:e^−^, H_2_ is incorporated into Ca_2_N:e^−^ as H^−^ ions to form Ca_2_NH (reaction 1). The H^−^ ions are released from Ca_2_NH, which leaves electrons to form a hydrogen vacancy near the Ru-support interface (reaction 2). The cleavage of N_2_ proceeds effectively on Ru surfaces due to electron injection from [Ca_2_N]^+^·e_1−*x*_^−^H_*x*_^−^ and the nitrogen adatoms react with H radicals to form ammonia (reaction 3).

Ammonia synthesis from N_2_ and D_2_ was examined to elucidate the reaction mechanism over Ru/[Ca_2_N]^+^·e_1−*x*_^−^H_*x*_^−^. The initial gas (reaction time = 0 h) consists mainly of N_2_ (*m*/*z* = 28) and D_2_ (*m*/*z* = 4), whereas the amounts of other species such as H_2_ (*m*/*z* = 2) and HD (*m*/*z* = 3) are negligibly small (Fig. S4†). As the reaction time increased, the N_2_ and D_2_ peaks decreased and the signals with *m*/*z* = 19, 18, 17, and 16 increased (Fig. 4a and S4 and S5†). These signals are attributed to ND_2_H, NDH_2_, ND_2_ (a fragment of ND_3_), NH_3_, and NH_2_ (a fragment of NH_3_). The signals with *m*/*z* = 17 and 16 are much larger than those with *m*/*z* = 18 and 19 in the early stage (0–3 h), which indicates that the dominant product from the reaction of N_2_ and D_2_ is NH_3_, rather than NH_2_D, NHD_2_, or ND_3_. Therefore, N adatoms react with H atoms derived from [Ca_2_N]^+^·e_1−*x*_^−^H_*x*_^−^ to form NH_3_, whereas D adatoms produced by the dissociative adsorption of D_2_ on Ru surfaces prefer to be incorporated into [Ca_2_N]^+^·e_1−*x*_^−^H_*x*_^−^ as D^−^ ions rather than directly react with N adatoms. TPD measurements (Fig. 4b) show that H_2_, HD, and D_2_ species were desorbed from the catalyst after the reaction. It is thus evident that some H^−^ ions in Ru/[Ca_2_N]^+^·e_1−*x*_^−^H_*x*_^−^ are replaced by D^−^ ions during the ammonia synthesis reaction. The incorporation of D^−^ ions proceeds simultaneously with NH_3_ formation, so that the exchange rate of H^−^ ions in Ru/[Ca_2_N]^+^·e_1−*x*_^−^H_*x*_^−^ with D^−^ ions can be estimated to be 1.66 mmol g^−1^ h^−1^ from the initial NH_3_ formation rate (0–3 h). On the other hand, when Ru/[Ca_2_N]^+^·e_1−*x*_^−^H_*x*_^−^ is heated at 340 °C under a D_2_ atmosphere without N_2_ (Fig. S6†), the exchange rate of H^−^ with D^−^ ions remains at only 0.18 mmol g^−1^ h^−1^, which is lower by an order of magnitude than that (1.66 mmol g^−1^ h^−1^) shown in Fig. 4a. These results reveal that N adatoms on Ru/[Ca_2_N]^+^·e_1−*x*_^−^H_*x*_^−^ preferentially react with H atoms derived from H^−^ ions in [Ca_2_N]^+^·e_1−*x*_^−^H_*x*_^−^ rather than H atoms produced by the dissociative adsorption of H_2_ on Ru surfaces because H atoms on Ru are readily incorporated into the support material to form H^−^ ions by reaction with an anionic electron.

To summarize these results, we propose the reaction mechanism illustrated in Fig. 4c. The dissociative adsorption of H_2_ forms H adatoms on Ru surfaces under the reaction conditions, and Ca_2_N:e^−^ is readily transformed into Ca_2_NH by the reaction of anionic electrons in Ca_2_N:e^−^ with spillover H adatoms ([Ca_2_N]^+^·e^−^ + H → [Ca_2_N]^+^·H^−^, reaction 1 in Fig. 4c). Ca_2_N:e^−^ and Ca_2_NH are in an equilibrium through the reversible hydrogen storage reaction; therefore, nonstoichiometric [Ca_2_N]^+^·e_1−*x*_^−^H_*x*_^−^ rather than stoichiometric Ca_2_NH is expected to result from the transformation (reaction 2 in Fig. 4c). [Ca_2_N]^+^·e_1−*x*_^−^H_*x*_^−^ with small work functions strongly donate electrons into Ru, which facilitates the cleavage of N_2_ molecules on Ru surfaces. N adatoms on Ru surfaces prefer to react with H atoms derived from the hydride to form ammonia and anionic electrons (hydrogen vacancies) in [Ca_2_N]^+^·e_1−*x*_^−^H_*x*_^−^ (reaction 3 in Fig. 4c), as demonstrated in the D_2_ experiments. This mechanism is distinct from ammonia synthesis on conventional heterogeneous catalysts, where the reaction between N and H adatoms on transition metal surfaces produces ammonia.^34^ The incorporation of hydrogen adatoms formed on Ru surfaces into the 2D layer of Ca_2_N is driven by reaction with anionic electrons and results in the suppression of active site saturation by hydrogen, *i.e.*, hydrogen poisoning of Ru. Thus, the reaction mechanism is very similar to that for Ru/C12A7:e^−^, as elucidated by kinetic analyses.^16^ Therefore, the rate-determining step for ammonia synthesis over Ru/Ca_2_N:e^−^ is not N_2_ cleavage, but subsequent processes, possibly the formation of NH species. However, Ru/Ca_2_N:e^−^ is far superior to Ru/C12A7:e^−^ in terms of catalytic performance for ammonia synthesis below 300 °C; Ru/C12A7:e^−^ has moderate catalytic activity and high activation energy (90 kJ mol^−1^) for ammonia synthesis below 300 °C, where the reaction mechanism is analogous to that for conventional catalysts. The H_2_ absorption–desorption characteristics of Ru/C12A7:e^−^ are observed above *ca.* 350 °C,^16^ which is due mainly to the stabilization of H^−^ ions in the positively charged sub-nanometer sized cages, so that a larger thermal energy is necessary to release hydrogen through the cage wall.^35^ On the other hand, the facile hydrogen exchange reaction on Ru/Ca_2_N:e^−^ proceeds at lower temperatures (from 200 °C) than that on Ru/C12A7:e^−^ because H^−^ ions are located in the open spaces between two cationic slabs [Ca_2_N]^+^. This facile hydrogen exchange at lower temperatures thus makes it possible for noticeable ammonia synthesis to occur, even at *ca.* 200 °C.

The experimental results described above demonstrate the validity of our proposed design concept for the Ru-support for low-temperature NH_3_ synthesis, *i.e.*, the reversible exchangeability between H^−^ ions and electrons plays an important role for effective ammonia synthesis at low temperatures. The effectiveness of this idea is further demonstrated by the use of CaH_2_, the simplest hydride, as a support for a Ru catalyst. Although CaH_2_ itself has a WF of 4.0 eV, the WF of CaH_2_ with V_H_ (*i.e.*, CaH_2−*x*_) has a small value (2.3 eV) similar to that for nonstoichiometric Ca_2_NH (Table 2). Ru/CaH_2_ exhibits high catalytic activity with a low activation energy (51 kJ mol^−1^), as expected from its low work function (Table 1), and has reaction orders for N_2_ and H_2_ similar to those for Ru/Ca_2_N:e^−^ (Table S1†). The formation of hydrogen vacancies in CaH_2_ is difficult because hydrogen desorption from CaH_2_ occurs above 600 °C.^36^ However, hydrogen can easily desorb from the surface of CaH_2_ above 200 °C in the presence of a Ru catalyst (Fig. S7†). Thus, anionic electrons are formed in Ru/CaH_2_ during the reaction, which results in efficient ammonia synthesis at lower reaction temperatures.

## Conclusions

Ru/Ca_2_N:e^−^ exhibits much higher catalytic performance for ammonia synthesis at low temperatures than heterogeneous catalysts reported to date, including Ru/C12A7:e^−^. This is not attributed to the Ca_2_N:e^−^ electride, but to a hydrogen-deficient Ca_2_NH hydride ([Ca_2_N]^+^·e_1−*x*_^−^H_*x*_^−^) formed during the ammonia synthesis reaction. Both Ru/CaH_2_ and Ru/Ca_2_NH hydrides exhibit high catalytic activity for ammonia synthesis. The formation of anionic electrons in these hydrides results in a small work function (2.3 eV), which accounts for the strong electron donation ability that facilitates efficient N_2_ cleavage on Ru. N adatoms on Ru preferentially react with H atoms derived from the hydride to form NH species. These reactions proceed even at *ca.* 200 °C, so that ammonia synthesis is catalyzed above 200 °C. In addition, the hydrides suppress H_2_ poisoning of the Ru surface due to their hydrogen storage properties. The present results demonstrate that the strong electron-donating ability and the reversible exchangeability between H^−^ ions in the lattice and anionic electrons at low temperatures are requisite for the Ru catalyst support in low-temperature ammonia synthesis.

## Supplementary Material

SC-007-C6SC00767H-s001
