# Correction: Essential role of hydride ion in ruthenium-based ammonia synthesis catalysts

**DOI:** 10.1039/c6sc90039a

**Published:** 2016-06-23

**Authors:** Masaaki Kitano, Yasunori Inoue, Hiroki Ishikawa, Kyosuke Yamagata, Takuya Nakao, Tomofumi Tada, Satoru Matsuishi, Toshiharu Yokoyama, Michikazu Hara, Hideo Hosono

**Affiliations:** a Materials Research Center for Element Strategy , Tokyo Institute of Technology , 4259 Nagatsuta, Midori-ku , Yokohama 226-8503 , Japan . Email: hosono@msl.titech.ac.jp; b Laboratory for Materials and Structures , Tokyo Institute of Technology , 4259 Nagatsuta, Midori-ku , Yokohama 226-8503 , Japan . Email: mhara@msl.titech.ac.jp; c ACCEL , Japan Science and Technology Agency , 4-1-8 Honcho , Kawaguchi , Saitama 332-0012 , Japan; d Frontier Research Center , Tokyo Institute of Technology , 4259 Nagatsuta, Midori-ku , Yokohama 226-8503 , Japan

## Abstract

Correction for ‘Essential role of hydride ion in ruthenium-based ammonia synthesis catalysts’ by Masaaki Kitano *et al.*, *Chem. Sci.*, 2016, **7**, 4036-4043.



## 

The authors regret that in the original article incorrect units were used to define both the number of surface Ru atoms (*N*_s_) and the ammonia synthesis rate (*r*_NH_3__) in columns 6 and 7 of Table 1. In both cases, ‘μmol’ should have been used instead of ‘mmol’. Therefore, the correct units for *N*_s_ and *r*_NH_3__ are ‘μmol g^–1^’ and ‘μmol g^–1^ h^–1^’ respectively.

The Royal Society of Chemistry apologises for these errors and any consequent inconvenience to authors and readers.

