# Supplementary material for: Essential role of hydride ion in ruthenium-based ammonia synthesis catalysts
Source: Chem Sci. 2016 Apr 21;7(7):4036–43. doi: 10.1039/c6sc00767h (PMC6013784; doi:10.1039/c6sc00767h)
Supplement: SC-007-C6SC00767H-s001 [file SC-007-C6SC00767H-s001.pdf]

Electronic Supplementary Material (ESI) for Chemical Science.
This journal is © The Royal Society of Chemistry 2016

Supporting Information for

Essential Role of Hydride Ion in Ruthenium-based Ammonia Synthesis Catalysts

Masaaki Kitano,[a] *Yasunori Inoue,*[b] *Hiroki Ishikawa,*[b] *Kyosuke Yamagata,*[b] *Takuya Nakao,*[b] *Tomofumi Tada,*[a] *Satoru Matsuishi,*[a] *Toshiharu Yokoyama,*[ac] *Michikazu Hara,*[bcd*] *and Hideo Hosono*[abcd*]

[a]Materials Research Center for Element Strategy, Tokyo Institute of Technology, 4259 Nagatsuta, Midori-ku, Yokohama 226-8503, Japan

[b]Laboratory for Materials and Structures, Tokyo Institute of Technology, 4259 Nagatsuta, Midori-ku, Yokohama 226-8503, Japan.

[c]ACCEL, Japan Science and Technology Agency, 4-1-8 Honcho, Kawaguchi, Saitama, 332-0012, Japan.

[d]Frontier Research Center, Tokyo Institute of Technology, 4259 Nagatsuta, Midori-ku, Yokohama 226-8503, Japan.

Experimental Section

Materials.

$Ca_2N:e^-$ powder was synthesized by solid-state reaction of Ca_3N_2 powder and Ca metal shot. Ca_3N_2 powder was first mixed with Ca metal shot at a molar ratio of 1:1 and the mixture was uniaxially pressed into a pellet form under pressure (20–30 MPa). The pellet was covered with molybdenum foil and sealed in an evacuated silica tube. The silica tube was heated at 800°C for 50 h in a vacuum, followed by quenching into water. The obtained sample was then ground into a powder under an Ar atmosphere. As a reference samples, Ca_2NH was synthesized by heating Ca_2N powder at 300°C in a flow of N_2 and H_2 (N_2:H_2 = 1:3, flow rate = 60 mL min^{-1}, pressure = 0.1 MPa), and CaNH was prepared by heating Ca_3N_2 at 600°C in a N_2 and H_2 gas mixture under the same flow conditions. XRD patterns of the obtained samples were consistent with the standard diffraction patterns (Fig. S8). CaH_2 was prepared by heating Ca metal at 400°C for 10 h in a H_2 atmosphere (2.0 MPa).

Ru-loading on $Ca_2N:e^-$, Ca_3N_2, and CaNH was conducted by the following procedure. The sample powder and $Ru_3(CO)_{12}$ were sealed in an evacuated silica tube and heated under the following temperature program: 2°C min^{-1} up to 40°C, hold for 1 h; up to 70°C over 2 h, hold for 1 h; up to 120°C over 2 h, hold for 1 h; and up to 250°C over 2.5 h, hold for 2 h; cooling down to ambient temperature.

Catalytic reaction.

Ammonia synthesis was conducted in a fixed bed flow system with a synthesis gas (H_2:N_2 = 3:1) at a flow rate of 60 mL min^{-1}. The reaction temperature was varied from 200 to 340°C and the pressure was kept at 0.1 MPa. All kinetic experiments were conducted far from equilibrium conditions (for example, the conversion level was less than 30% of that at equilibrium). The reaction orders with respect to N_2 and H_2 were obtained at a constant flow rate (60 ml min^{-1}) using Ar gas as a diluent, and that for NH_3 was determined with ($3H_2$+N_2) by changing the synthesis gas flow rate. The produced ammonia was trapped by in a 5 mM sulfuric acid solution and the amount of NH_4^+ generated in the solution was determined using ion chromatography (LC-2000 plus, Jasco). Ammonia synthesis from N_2 and D_2 was conducted using a U-shaped glass reactor connected to a closed gas circulation system. The mixture of N_2 and D_2 gases (total pressure: 60 kPa, N_2:D_2 = 1:3) was introduced into the glass system. The change in the composition of the circulating gas was monitored with a quadrupole mass spectrometer (M-101QA-TDM, Canon Anelva Corp.) and Ar was used as a carrier gas. The circulating pump placed in the system removes diffusional and adsorption/desorption limitations. The m/z = 2, 3, 4, 16, 17, 18, 19, 20, and 28 masses were monitored as a function of time to follow the reaction. ND_3 (m/z = 20) was hardly detectable because it overlaps with a fragment of Ar (m/z =20). The fragmentation factor of m/z = 20, 40 for Ar is 0.12, 0.88, respectively.

Characterization.

N_2 adsorption-desorption isotherms were measured at -196°C using a specific surface area analyzer (Nova 4200e, Quantachrome) after evacuation of the sample at 300°C. The Ru dispersion, mean particle size, and the number of surface Ru atoms were determined by CO pulse chemisorption at 50°C with a He flow of 30 mL min^{-1} and 0.09 mL pulses of 9.88% CO in He using a catalyst analyzer (BELCAT-A, MicrotracBEL, Japan); a stoichiometry of Ru/CO = 1 was assumed. The

crystal structure was identified using XRD (D8 Advance, Bruker) with monochromated Cu Kα radiation (λ = 0.15418 nm). Raman spectra of the samples were measured with a spectrometer (HR-800, Horiba Jobin Yvon Co. Ltd., Japan) using a laser with a wavelength of 457.4 nm. TPA of H_2 measurements were conducted by heating (1°C min^{-1}) a sample (ca. 100 mg) in a stream of 4.8% H_2/Ar mixture, and the consumption of H_2 was monitored with a thermal conductivity detector (TCD) and mass spectrometer (BELMass, MicrotracBEL, Japan). TPD of H_2 was performed using the same instrument as TPA experiment. Prior to the TPD measurements, the sample was heated under a mixture of H_2 and N_2, H_2:N_2 = 3:1, 60 mL min^{-1}, 0.1 MPa, 340°C, 10 h, which are the same reaction conditions as those used for ammonia synthesis. After cooling to room temperature, the sample was placed into a TPD glass reactor in an Ar-filled glovebox. The sample was then heated (1°C min^{-1}) in an Ar stream (30 mL min^{-1}), and the concentration of H_2 was monitored with a thermal conductivity detector (TCD) and a mass spectrometer (BELMass, MicrotracBEL, Japan).

Supplementary Results

Table S1 Orders of reaction for ammonia synthesis over various Ru catalysts.

Catalyst	$\alpha(N_2)$	$\beta(H_2)$	$\gamma(NH_3)$
Ru/Ca_2N	0.53	0.79	-1.03
Ru/CaNH	0.77	-0.19	-0.72
Ru/CaH_2	0.55	0.87	-1.11
Ru/C12A7:e^-	0.46	0.97	-1.00
Ru/C12A7:O^{2-}	1.00	0	-0.25
Ru-Cs/MgO	1.0	-0.45	-0.37

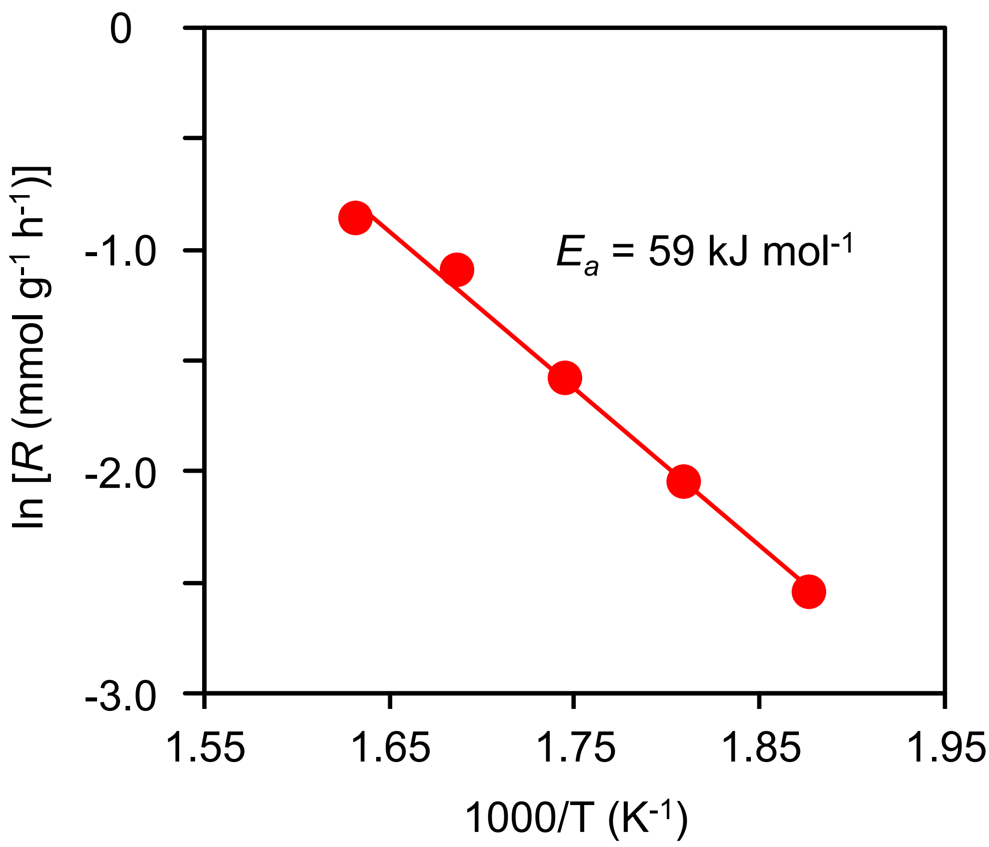

Figure S1 Temperature dependence of the rate (*R*) of N_2 isotopic exchange reaction over Ru/Ca_2N:e⁻ at 26.7 kPa ($^{15}N_2$: $^{14}N_2$ = 1 : 4). E_a is the apparent activation energy calculated from Arrhenius plots of the reaction rate in the temperature range of 260-340ºC. Before the reaction, Ru/Ca_2N:e⁻ was pretreated under under N_2 and H_2 flow (N_2: H_2 = 1:3) at 340ºC for 24 h.

The activation energy (59 kJ mol^{-1}) of Ru/Ca_2N:e⁻ is much smaller than that of conventional Ru catalysts (>130 kJ mol^{-1}) reported in the previous literature [S1] and is comparable to that of Ru/C12A7:e⁻ (58 kJ mol^{-1}).

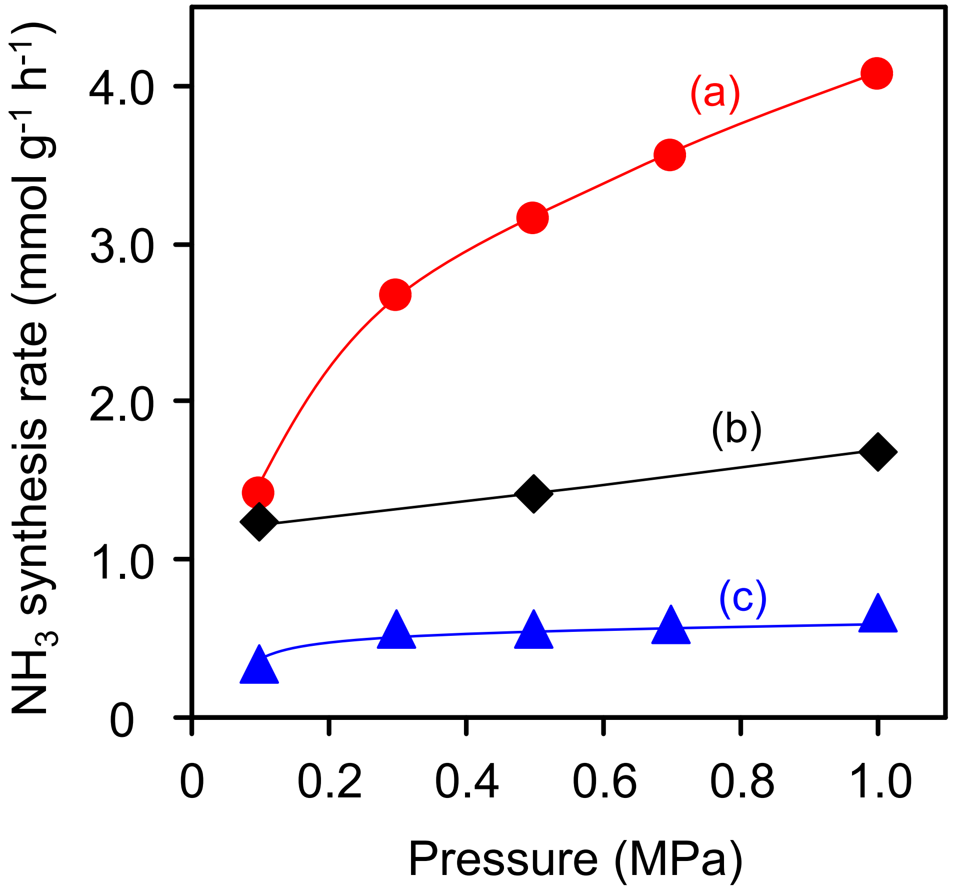

Figure S2 Pressure dependence of the rate of ammonia synthesis over (a) Ru/$Ca_2N:e^-$, (b) Ru/C12A7:e^-, (c) Ru/CaNH. (Reaction conditions: catalyst, 0.1 g; WHSV, 36000 mL g_{cat}^{-1} h^{-1}; reaction temperature, 320ºC).

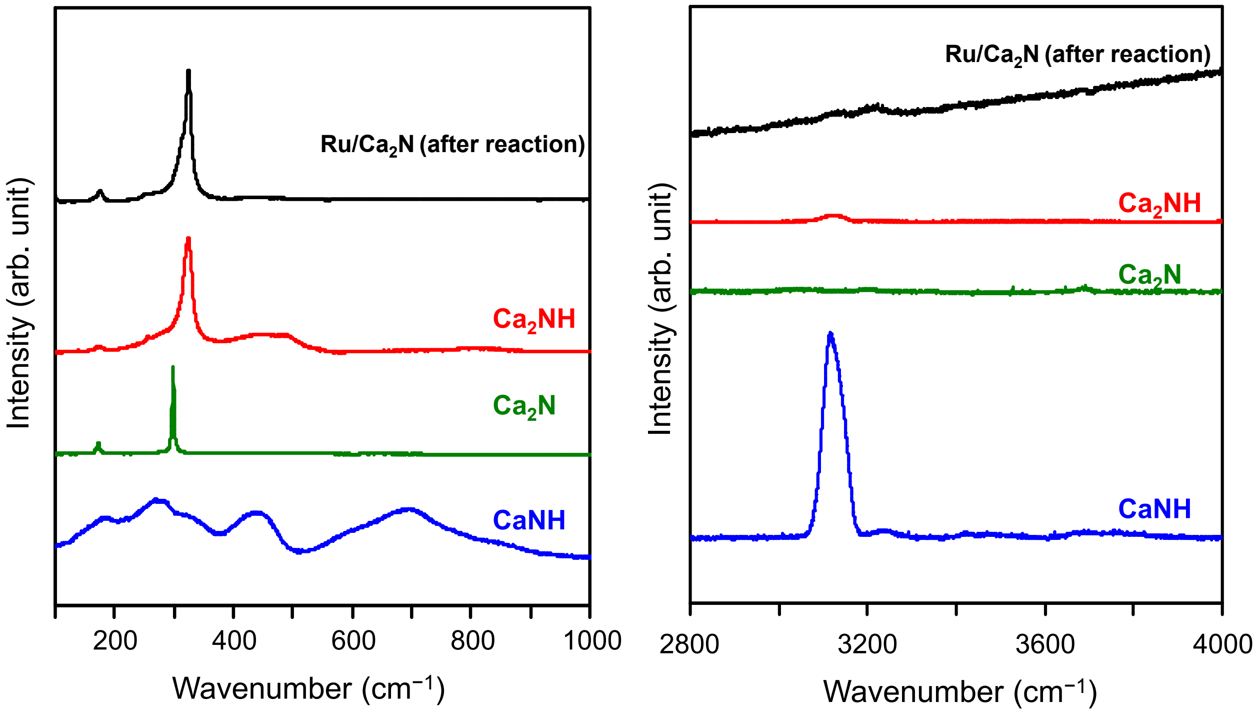

Figure S3 Raman spectra of Ru/Ca_2N:e^- after ammonia synthesis reaction at 340ºC for 20 h together with the spectra of CaNH, Ca_2N, and Ca_2NH.

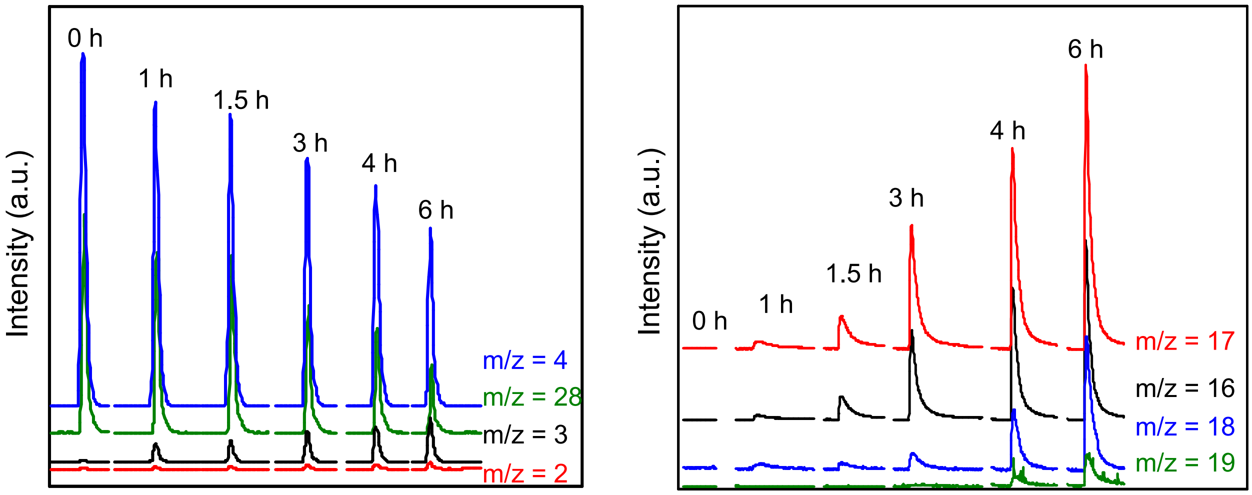

Figure S4 Mass spectra for ammonia synthesis over Ru/Ca_2N:e^-. Reaction conditions: catalyst, 0.2 g; reaction temperature, 340ºC; reaction gas, N_2:D_2 = 1:3; reaction pressure, 60 kPa.

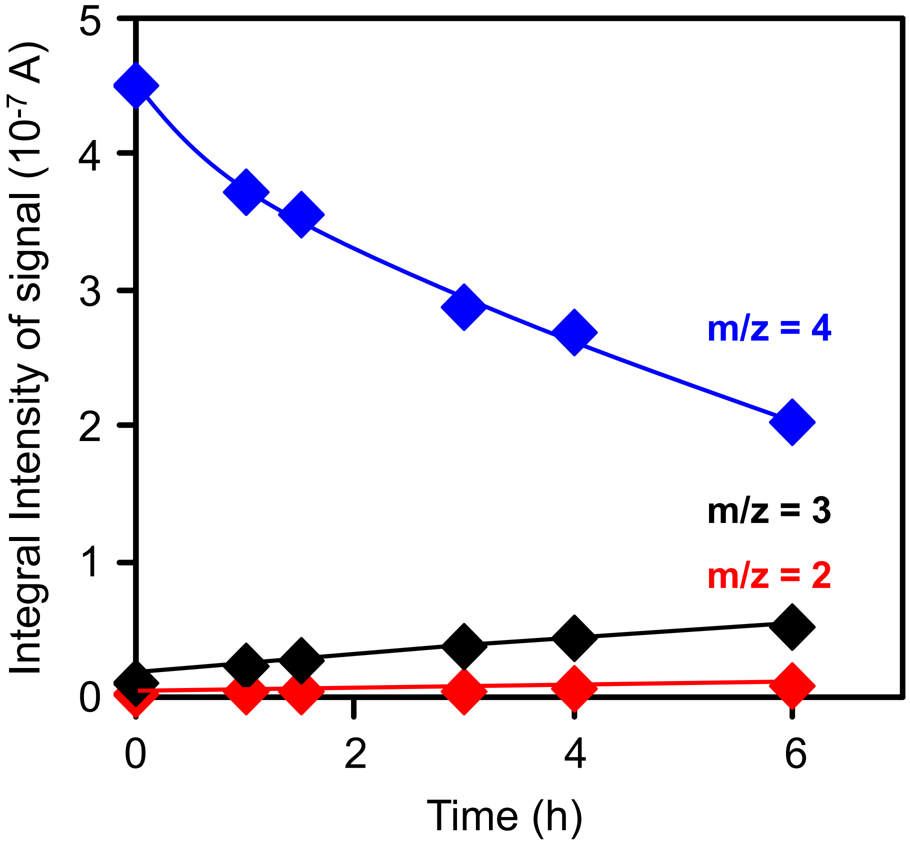

Figure S5 Reaction time profiles of H_2 (m/z =2), D_2 (m/z =4) and HD (m/z =3) during ammonia synthesis from N_2 and D_2 over Ru/Ca_2N:e^-. Before the reaction, Ru/Ca_2N:e^- was heated under N_2+H_2 flow (N_2:H_2 = 1:3) at 340ºC for 10 h. Then, the obtained catalyst was heated under N_2 + D_2 atmosphere. (Reaction conditions: catalyst, 0.2 g; reaction temperature, 340ºC; reaction gas, N_2:D_2 = 1:3; reaction pressure, 60 kPa).

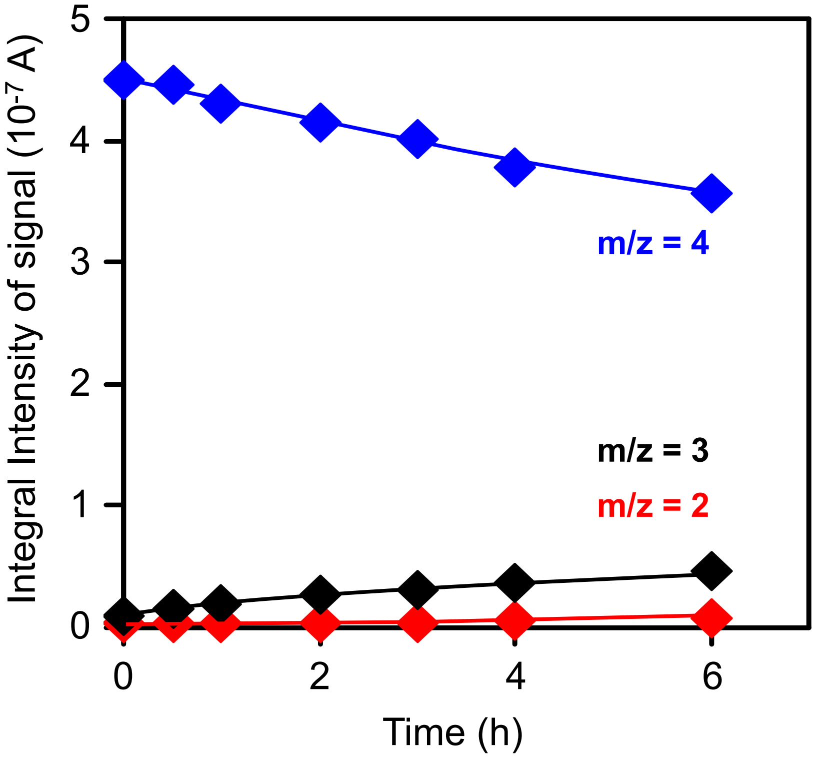

Figure S6 Reaction time profiles of hydrogen exchange over Ru/Ca_2N:e^-. Before the reaction, Ru/Ca_2N:e^- was heated under N_2+H_2 flow (N_2:H_2 = 1:3) at 340ºC for 10 h. Then, the obtained catalyst was heated under D_2 atmosphere without N_2, where the partial pressure of D_2 was adjusted to the same value as the case of N_2 + D_2 system (Fig. 4b) by adding Ar gas. (Reaction conditions: catalyst, 0.2 g; reaction temperature, 340ºC; reaction gas, Ar:D_2 = 1:3; reaction pressure, 60 kPa).

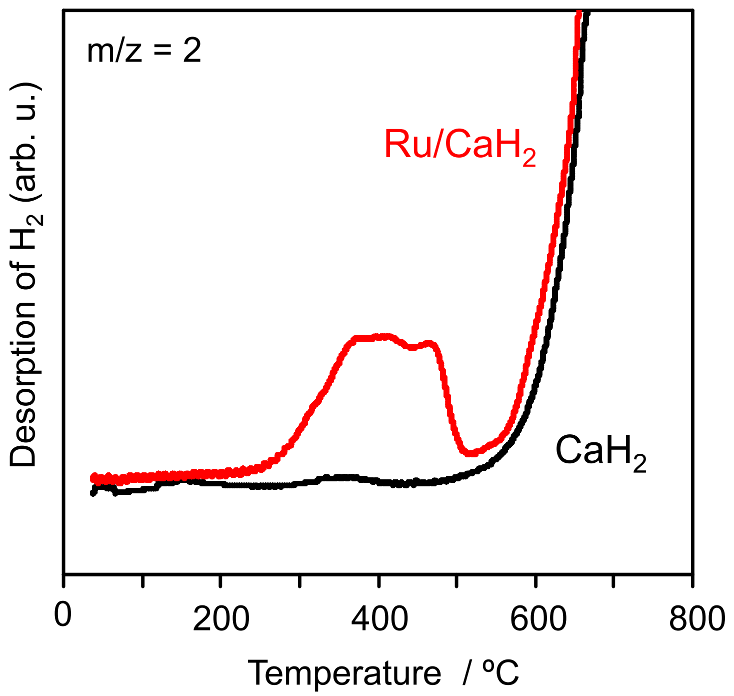

Figure S7 H_2 TPD profiles of Ru/CaH_2 and CaH_2. The TPD experiment was performed (1°C min^{-1}) under Ar flow.

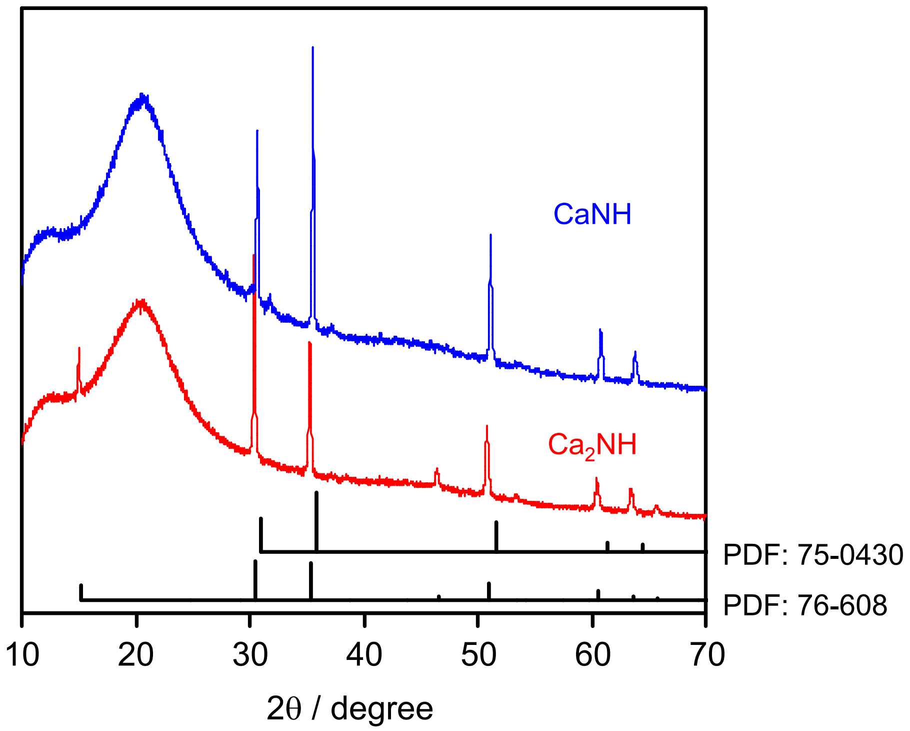

Figure S8 X-ray diffraction patterns of Ca_2NH and CaNH. Standard JCPDS diffraction patterns for CaNH (PDF: 75-0430) and Ca_2NH (PDF: 76-608) are provided for reference.

Detail conditions for DFT calculations

Table S2 Computational conditions for DFT calculations.

Compound	*Unit Cell*	*Space Group*	*Surface index*	*Kpoints*	E^{cut}	*Vaccum width*
Ca_2N:e-	18(Ca_2N)	$R\bar{3}m$	(111)	7×7×1	520	20
Ca_2N:e-	18(Ca_2N)	$R\bar{3}m$	(112)	14×2×1	520	20
Ca_2NH	32(Ca_2NH)	$Fd\bar{3}m$	(100)	3×3×1	470	20
$Ca_2NH_{1-1/16}$	32($Ca_2NH_{1-1/16}$)	$Fd\bar{3}m$	(100)	3×3×1	470	20
$Ca_2NH_{1-2/16}$	32($Ca_2NH_{1-2/16}$)	$Fd\bar{3}m$	(100)	3×3×1	470	20
CaH_2	24(CaH_2)	Pnma	(010)	2×4×1	500	20
CaH_2	24($CaH_{2-1/12}$)	Pnma	(010)	2×4×1	500	20
CaNH	12(CaNH)	$Fm\bar{3}m$	(100)	5×5×1	500	—
Ru	12Ru	$P6_3/mmc$	(0001)	8×8×1	550	25
Ru/Ca_2NH	Ru_6/16(Ca_2NH)	$P6_3/mmc$	(100)	3×3×1	470	20
Ru/$Ca_2NH_{1-n/16}$	Ru_6/16($Ca_2NH_{1-n/16}$)	$P6_3/mmc$	(100)	3×3×1	470	20
Ru/$Ca_2NH_{1-n/16}$	Ru_{10}/16($Ca_2NH_{1-n/16}$)	$P6_3/mmc$	(100)	3×3×1	470	20

Vienna ab initio simulation package (VASP) [S2,S3] was adopted for structural relaxation and calculations for total energies and work functions. The projector-augmented wave (PAW) [S4,S5] method was used, and the electron exchange-correlation was described in Perdew-Burke- Ernzerhof type [S6] generalized gradient approximation. E^{cut} means cutoff energies for wave functions. Monkhorst-Pack k-point grids [S7] for the first Brillouin zone sampling are listed in *Kpoints* column. Note that the space groups correspond to those in the bulk condition, and that the normal vector of each surface is defined as the c-vector in *Kpoints* colum. The convergence criteria of energy and force are respectively 1.0×10^{-6} eV and 1.0×10^{-2} eV/Å.

Table S3 Computational models for Ru-loaded Ca_2NH with/without H-vacancies and N_2 molecule, and calculated formation energies of H-vacancies with respect to the stoichiometric Ru-loaded Ca_2NH. $\Delta E(V_H)$ of compound AH_{1-x} is the total energy difference defined as $[E(AH_{1-x}) + xE(H_2)/2]–E(AH)$, where $E(AH)$, $E(AH_{1-x})$, and $E(H_2)$ are respectively the total energies of the stoichiometric AH, H-deficient AH_{1-x}, and hydrogen molecule.

Model index	Unit Cell	$\Delta E(V_H)$ in eV
1	$Ru_6/16(Ca_2NH)$	—
2	$Ru_6/16(Ca_2NH_{1-1/16})$	0.673
3	$Ru_6/N_2/16(Ca_2NH)$	—
4	$Ru_6/N_2/16(Ca_2NH_{1-1/16})$	0.869
5	$Ru_6/N_2/16(Ca_2NH_{1-1/16})$	0.493
6	$Ru_6/N_2/16(Ca_2NH_{1-1/16})$	0.910
7	$Ru_6/N_2/16(Ca_2NH_{1-1/16})$	0.668
8	$Ru_{10}/16(Ca_2NH)$	—
9	$Ru_{10}/16(Ca_2NH_{1-1/16})$	0.431
10	$Ru_{10}/N_2/16(Ca_2NH_{1-1/16})$	0.663

Model 1

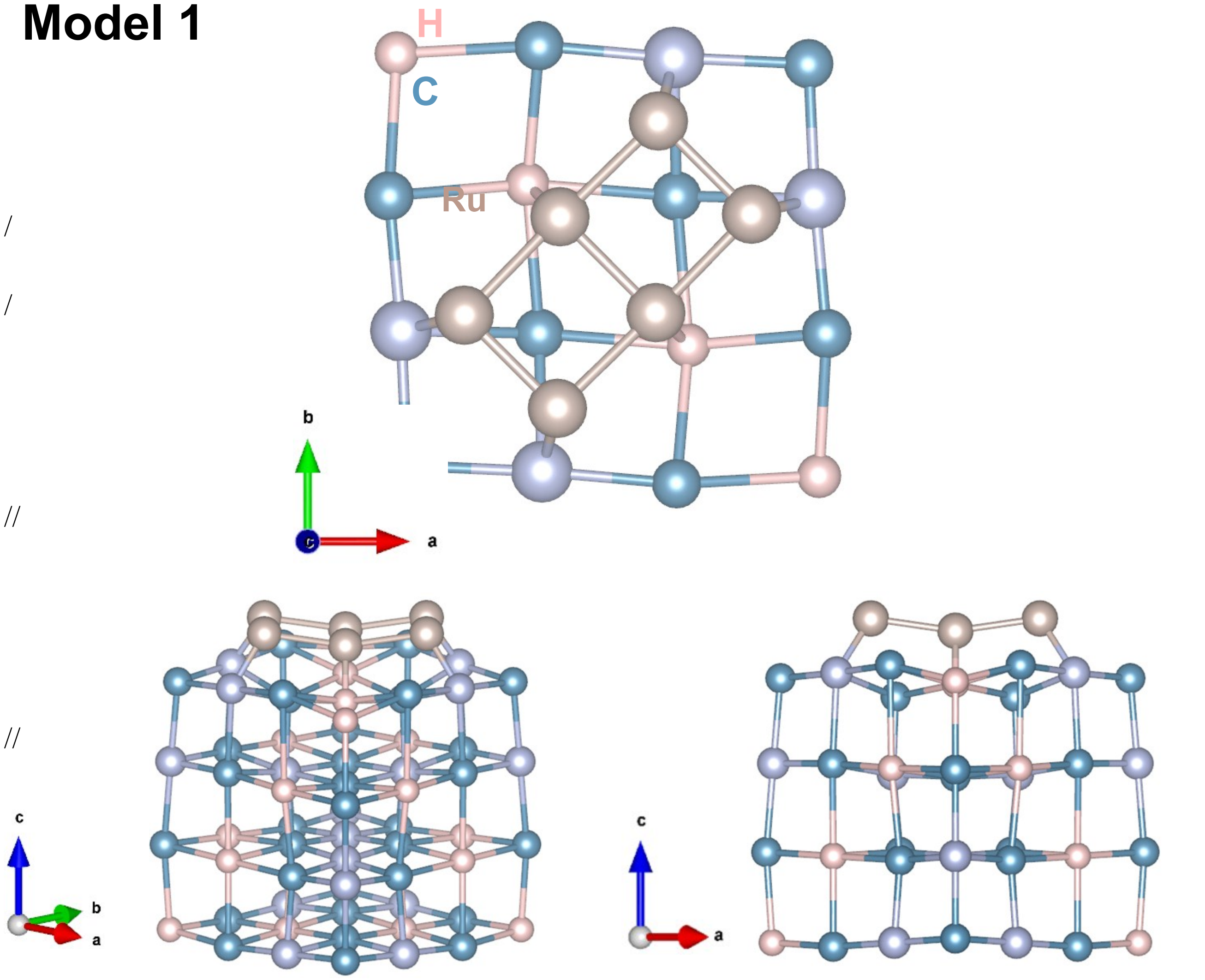

Figure S9 Computational models for Ru_6-loaded $Ca_2NH(100)$, Model 1 in Supplementary Table 4; top view (top) and side views (bottom two). The Ru_6 cluster and the topmost layer of $Ca_2NH(100)$ are depicted in the top view. Hydrogen atom indicated with dotted circles in the top view is the surface hydrogen removed in Ru-loaded Ca_2NH_{1-x} (100). Note the normal vector the surface is defined as the c-vector in the drawing. Visualization of the models was performed with VESTA [S8].

///

Model 2

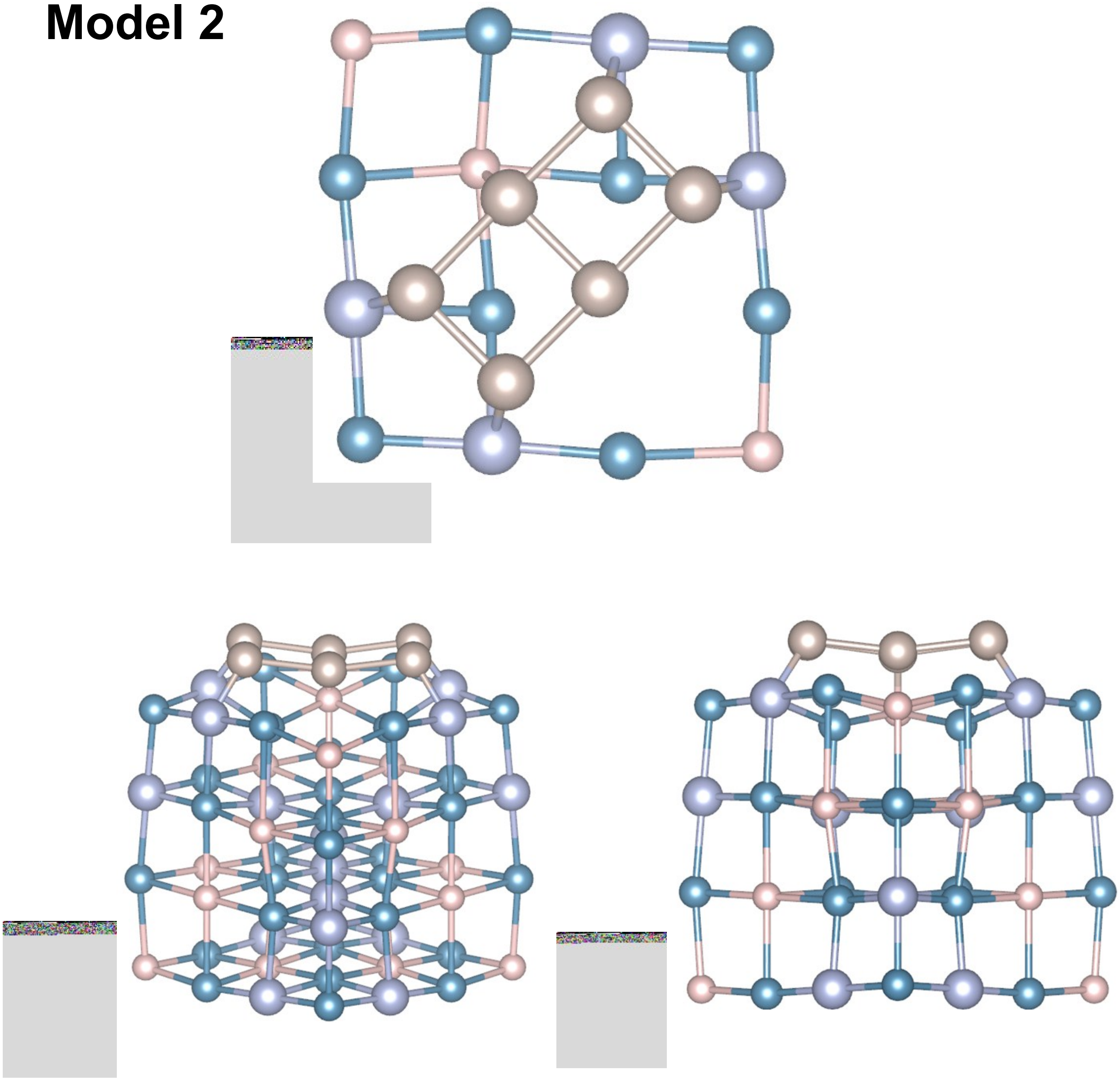

Figure S10 Computational models for Ru_6-loaded Ca_2NH_{1-x}(100), Model 2 in Supplementary Table 4; top view (top) and side views (bottom two). The Ru_6 cluster and the topmost layer of Ca_2NH_{1-x} (100) are depicted in the top view. Note the normal vector the surface is defined as the c-vector in the drawing.

Model 4

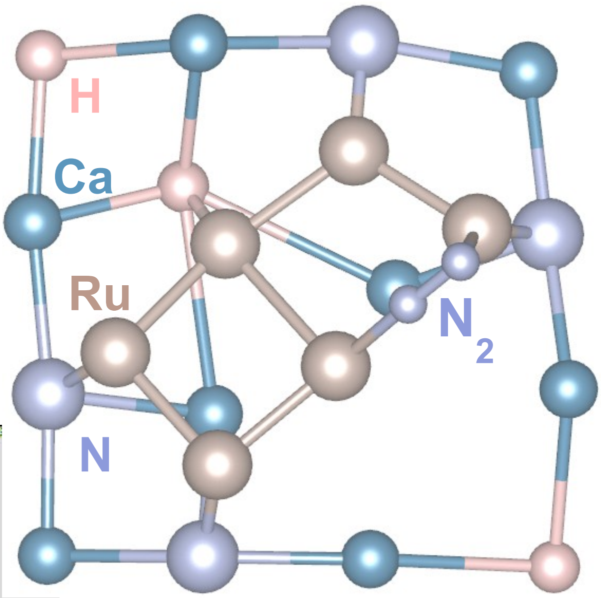

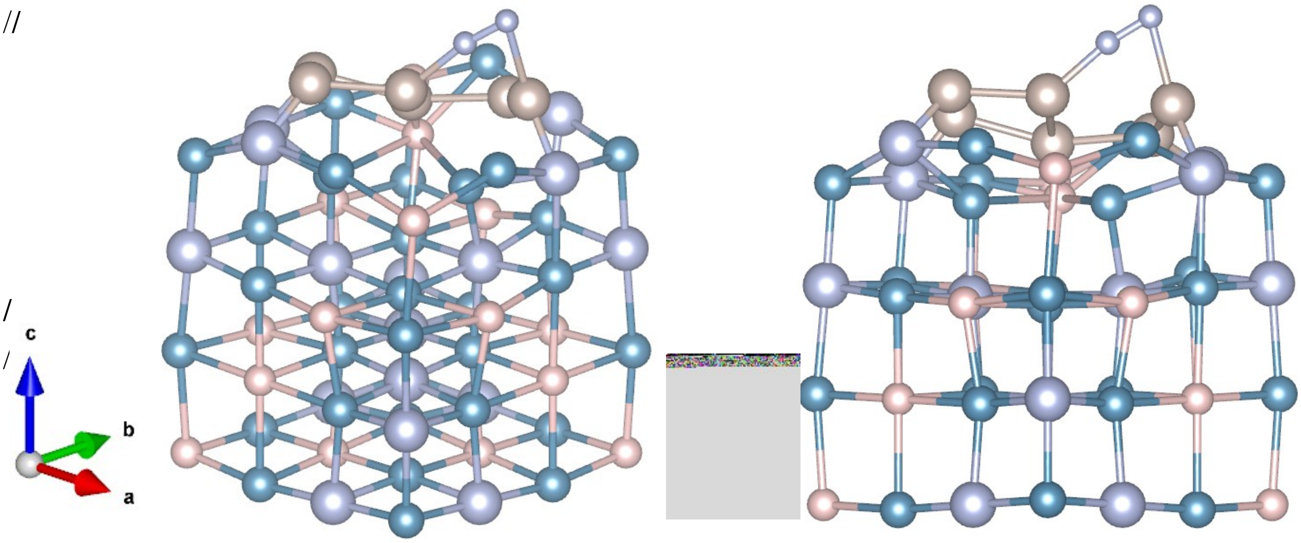

Figure S11 Computational models for Ru_6-loaded N_2-adsorbed Ca_2NH_{1-x}(100), Model 4 in Supplementary Table 4; top view (top) and side views (bottom two). The Ru_6 cluster, adsorbed N_2 molecule, and the topmost layer of Ca_2NH_{1-x} (100) are depicted in the top view. Note the normal vector the surface is defined as the c-vector in the drawing.

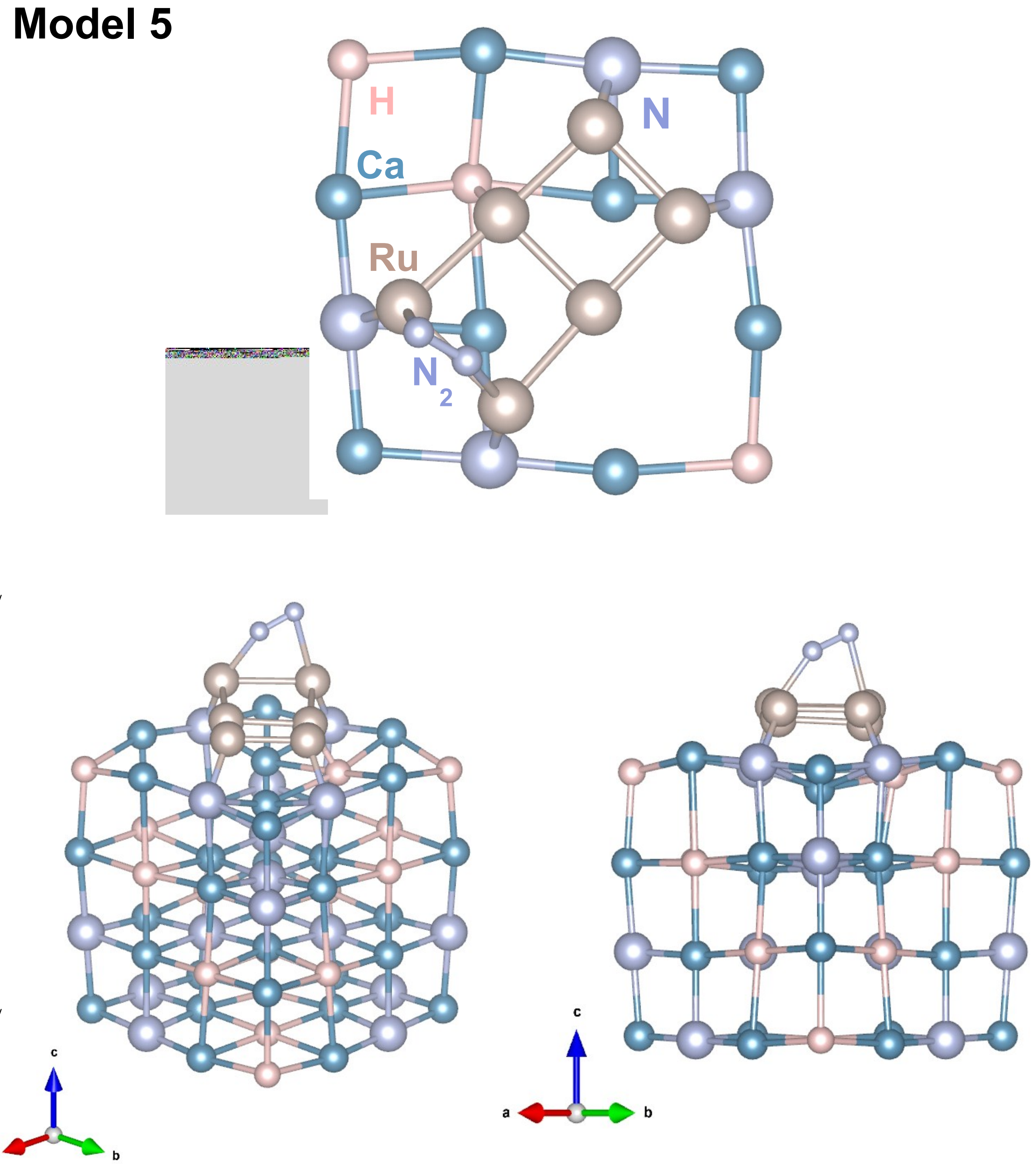

Figure S12 Computational models for Ru_6-loaded and N_2-adsorbed Ca_2NH_{1-x}(100), Model 5 in Supplementary Table 5; top view (top) and side views (bottom two). The Ru_6 cluster, adsorbed N_2 molecule, and the topmost layer of Ca_2NH_{1-x} (100) are depicted in the top view. Note the normal vector the surface is defined as the c-vector in the drawing.

Model 6

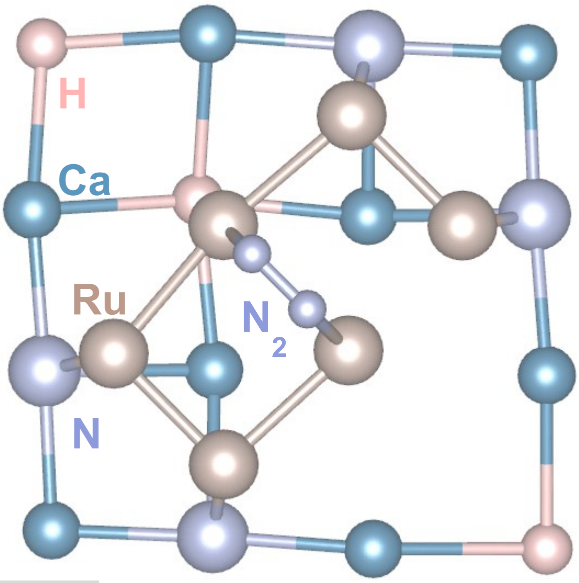

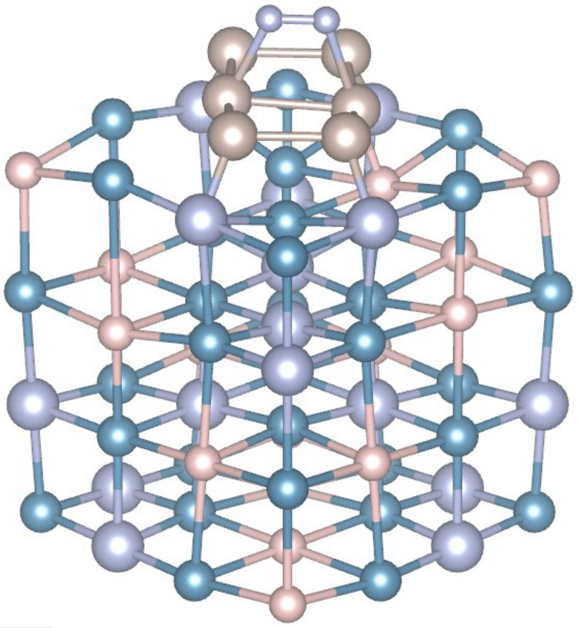

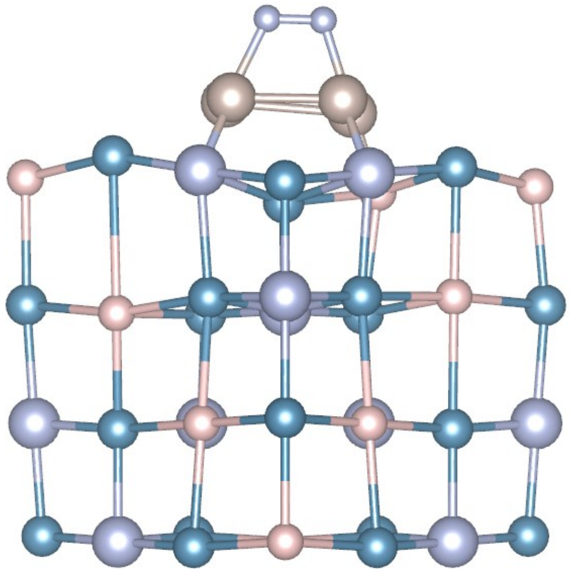

Figure S13 Computational models for Ru_6-loaded and N_2-adsorbed Ca_2NH_{1-x}(100), Model 6 in Supplementary Table 4; top view (top) and side views (bottom two). The Ru_6 cluster, adsorbed N_2 molecule, and the topmost layer of Ca_2NH_{1-x} (100) are depicted in the top view. Note the normal vector the surface is defined as the c-vector in the drawing.

Model 7

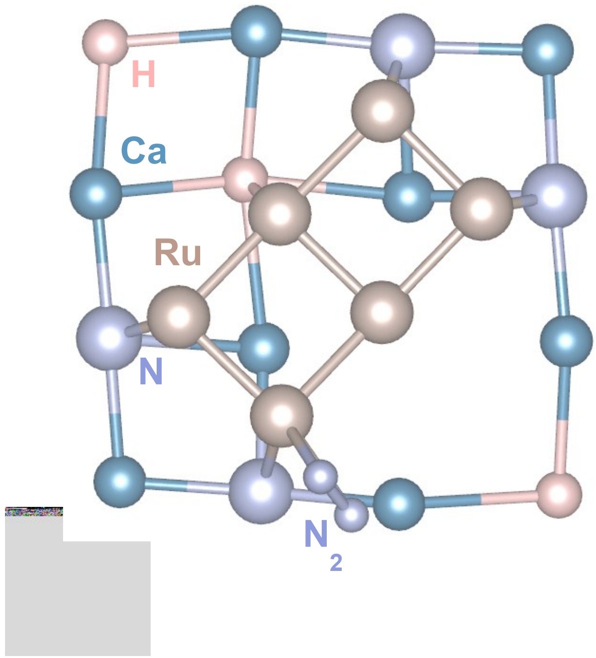

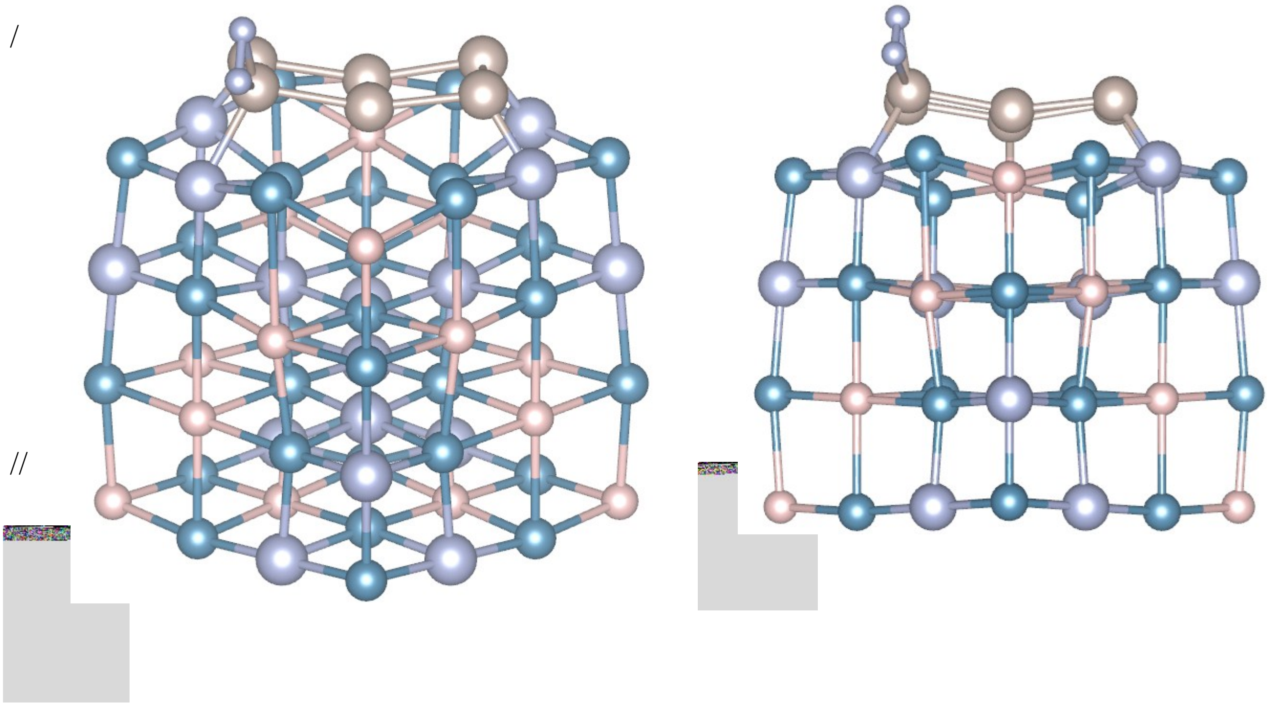

Figure S14 Computational models for Ru_6-loaded and N_2-adsorbed Ca_2NH_{1-x}(100), Model 7 in Supplementary Table 4; top view (top) and side views (bottom two). The Ru_6 cluster, adsorbed N_2 molecule, and the topmost layer of Ca_2NH_{1-x} (100) are depicted in the top view. Note the normal vector the surface is defined as the c-vector in the drawing.

Model 9

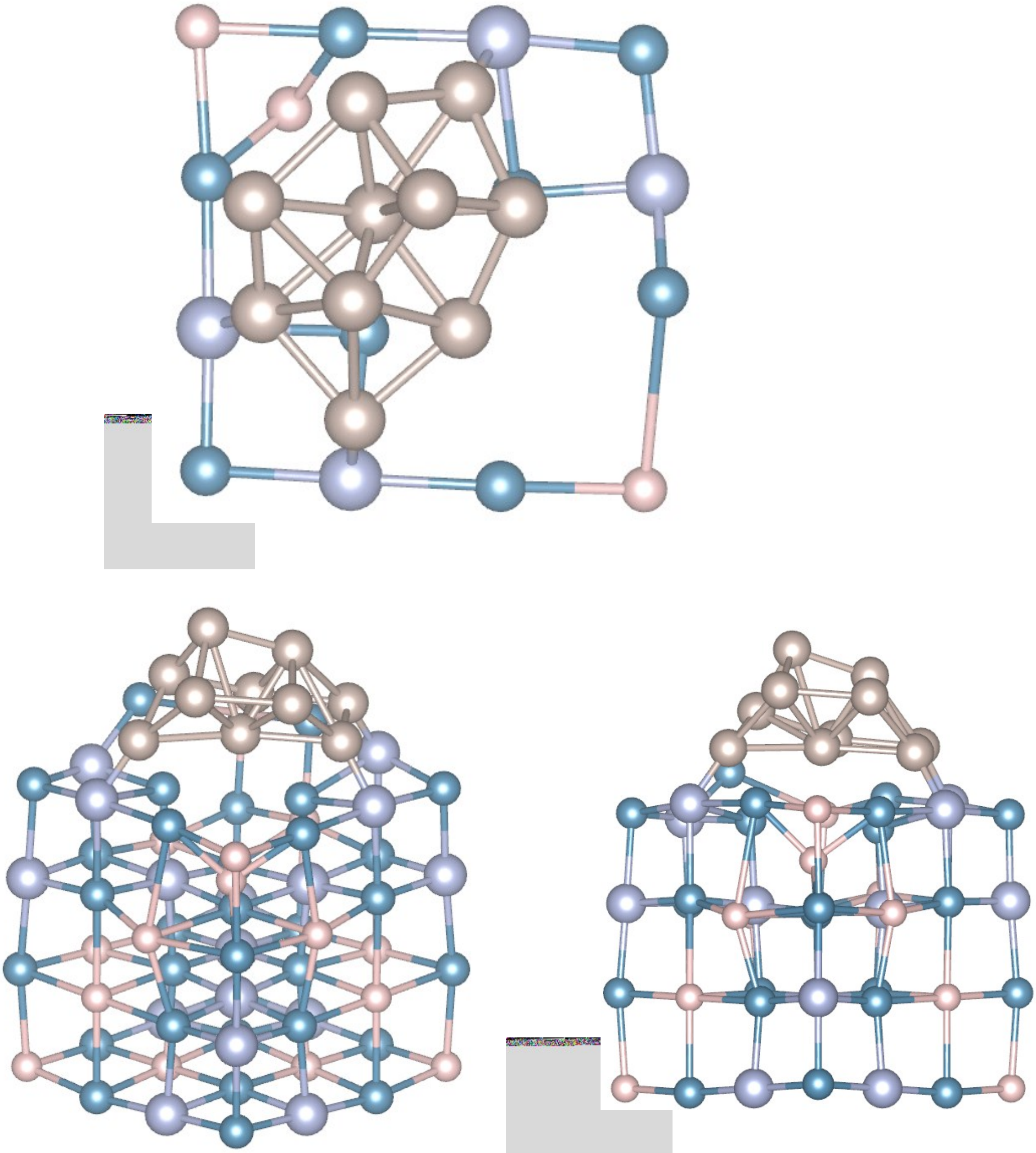

Figure S15 Computational models for Ru_{10}-loaded and N_2-adsorbed Ca_2NH_{1-x}(100), Model 9 in Supplementary Table 4; top view (top) and side views (bottom two). The Ru_{10} cluster, adsorbed N_2 molecule, and the topmost layer of Ca_2NH_{1-x} (100) are depicted in the top view. Note the normal vector the surface is defined as the c-vector in the drawing.

//

Model 10

/

////

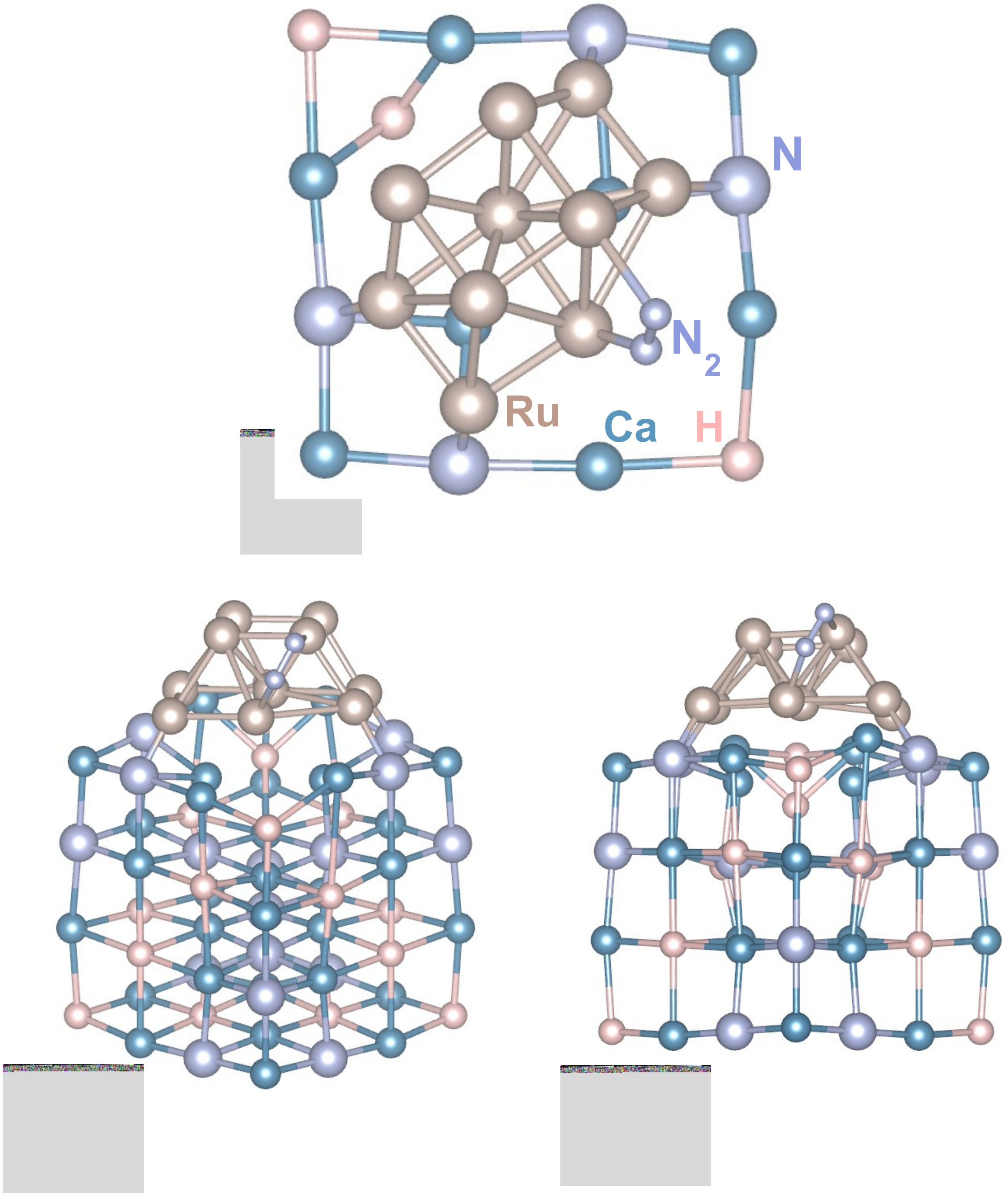

Figure S16 Computational models for Ru_{10}-loaded and N_2-adsorbed Ca_2NH_{1-x}(100), Model 10 in Supplementary Table 4; top view (top) and side views (bottom two). The Ru_{10} cluster, adsorbed N_2 molecule, and the topmost layer of Ca_2NH_{1-x} (100) are depicted in the top view. Note the normal vector the surface is defined as the c-vector in the drawing.

References in Supplementary Information

[S1] M. Kitano, et al., Electride support boosts nitrogen dissociation over ruthenium catalyst and shifts the bottleneck in ammonia synthesis, Nat Commun. 6, 6731, (2015).
[S2] G. Kresse, J. Furthmüller, Efficiency of ab-initio total energy calculations for metals and semiconductors using a plane-wave basis set, Comput. Mat. Sci., 6, 15-50 (1996).
[S3] G. Kresse, J. Furthmüller, Efficient iterative schemes for ab initio total-energy calculations using a plane-wave basis set, Phys. Rev. B, 54, 11169-11186 (1996).
[S4] P. E. Blöchl, Projector augmented-wave method, Phys. Rev. B 50, 17953-17979 (1994).
[S5] G. Kresse, D. Joubert, From ultrasoft pseudopotentials to the projector augmented-wave method, Phys. Rev. B 59, 1758-1775 (1999).
[S6] J. P. Perdew, K. Burke, M. Ernzerhof, Generalized Gradient Approximation Made Simple, Phys. Rev. Lett. 77, 3865-3868 (1996).
[S7] H. J. Monkhorst, J. D. Pack, Special points for Brillouin-zone integrations, Phys. Rev. B 13, 5188-5192 (1976).
[S8] K. Momma, F. Izumi, VESTA 3 for three-dimensional visualization of crystal, volumetric and morphology data, J. Appl. Crystallogr. 44, 1272-1276 (2011).
